# Mapping the Oxford Shoulder Score onto the EQ-5D utility index

**DOI:** 10.1007/s11136-022-03262-4

**Published:** 2022-09-28

**Authors:** Epaminondas M. Valsamis, David Beard, Andrew Carr, Gary S. Collins, Stephen Brealey, Amar Rangan, Rita Santos, Belen Corbacho, Jonathan L. Rees, Rafael Pinedo-Villanueva

**Affiliations:** 1 Nuffield Department of Orthopaedics, Rheumatology and Musculoskeletal Sciences, Botnar Research Centre, University of Oxford, Oxford, OX3 7LD UK; 2 Nuffield Department of Orthopaedics, Rheumatology and Musculoskeletal Sciences, Centre for Statistics in Medicine, University of Oxford, Oxford, OX3 7LD UK; 3 Department of Health Sciences, York Trials Unit, University of York, York, YO10 5DD UK; 4 Department of Health Sciences, York Trials Unit, University of York, York, YO10 5DD UK; 5 Centre for Health Economics, University of York, York, YO10 5DD UK

**Keywords:** Mapping, Oxford shoulder score, EQ-5D, Shoulder outcomes

## Abstract

**Purpose:**

In order to enable cost-utility analysis of shoulder pain conditions and treatments, this study aimed to develop and evaluate mapping algorithms to estimate the EQ-5D health index from the Oxford Shoulder Score (OSS) when health outcomes are only assessed with the OSS.

**Methods:**

5437 paired OSS and EQ-5D questionnaire responses from four national multicentre randomised controlled trials investigating different shoulder pathologies and treatments were split into training and testing samples. Separate EQ-5D-3L and EQ-5D-5L analyses were undertaken. Transfer to utility (TTU) regression (univariate linear, polynomial, spline, multivariable linear, two-part logistic-linear, tobit and adjusted limited dependent variable mixture models) and response mapping (ordered logistic regression and seemingly unrelated regression (SUR)) models were developed on the training sample. These were internally validated, and their performance evaluated on the testing sample. Model performance was evaluated over 100-fold repeated training–testing sample splits.

**Results:**

For the EQ-5D-3L analysis, the multivariable linear and splines models had the lowest mean square error (MSE) of 0.0415. The SUR model had the lowest mean absolute error (MAE) of 0.136. Model performance was greatest in the mid-range and best health states, and lowest in poor health states.

For the EQ-5D-5L analyses, the multivariable linear and splines models had the lowest MSE (0.0241–0.0278) while the SUR models had the lowest MAE (0.105–0.113).

**Conclusion:**

The developed models now allow accurate estimation of the EQ-5D health index when only the OSS responses are available as a measure of patient-reported health outcome.

**Supplementary Information:**

The online version contains supplementary material available at 10.1007/s11136-022-03262-4.

## Plain English summary

Collecting patient-reported outcome scores (PROMs) after shoulder surgery is a very important way of judging the success of surgery and identifying which healthcare treatments might be best for patients. PROMs such as the Oxford Shoulder Score (OSS) are now collected routinely around the world, especially by national joint registries. While the OSS is a valuable commonly used tool, a different type of questionnaire, such as the EQ-5D, is needed if we wish to know which shoulder treatments offer the best value for money. This is important for national health services to plan and deliver care. However, national shoulder registries do not routinely collect the EQ-5D alongside the OSS. In this study, we used questionnaire data from patients who took part in four national multicentre research studies called randomised trials. These studies assessed different types of surgical treatment for different types of shoulder problems, and patients completed both the OSS and EQ-5D questionnaires at the same time. This allowed us to compare the OSS to the EQ-5D using statistical modelling to see if we can predict a patient’s EQ-5D score from their OSS. We found that several statistical models were able to successfully do this. This means that from now on, researchers can use the large amounts of OSS data already collected and stored in national joint registries to predict EQ-5D scores and so draw important conclusions about the value for money of different surgical shoulder treatments.

## Introduction

Shoulder pain is associated with increased health care utilisation and accounts for 20% of disability claims for musculoskeletal disorders [1, 2]. Degenerative shoulder osteoarthritis causes pain, functional limitation and disability. Although difficult to accurately ascertain, shoulder osteoarthritis is estimated to have a prevalence between 4 and 26%, depending on the mode of diagnosis and population cohort [3–5]. Shoulder replacement is the established surgical technique for treating patients with end-stage shoulder arthritis, pain and disability. The global incidence of shoulder replacements is rising at a rapid rate with some countries reporting up to a 17- fold increase over the last 10 years [6].

While shoulder replacements have been shown to be effective at improving shoulder pain and function caused by joint arthritis, routinely collected patient-reported outcome measures (PROMs) suggest some patients may not benefit from the procedure, and the risk of serious adverse events following shoulder replacement may be higher than previously thought [7, 8]. Cost-utility studies are urgently required to appraise the different types of available shoulder replacements. This requires preference-based measures of utility such as the EQ-5D. There is no national PROMS programme in the United Kingdom (UK) that mandates or collects shoulder PROMs or the EQ-5D. The National Joint Replacement (NJR) registry in the UK is one of the largest and most complete joint registries in the world. However, the NJR does not collect EQ-5D from patients undergoing shoulder replacement; instead, it collects the Oxford Shoulder Score (OSS) which is not a health utility measure nor is it preference-based. The lack of any published mapping methods to estimate health utility from patients’ OSS score has precluded shoulder replacement cost-utility analyses and resulted in the National Institute for Health and Care Excellence (NICE) identifying no published cost-effectiveness studies to inform their most recent national guidelines [9]. The OSS is used in a wide range of shoulder conditions and in several different countries. Therefore, a mapping method to estimate health utility would enable cost-utility analyses in a range of different shoulder conditions in varying healthcare settings [10–12].

The primary objective of this study was to develop and assess the performance of different mapping algorithms to estimate the EQ-5D-3L health utility index from OSS responses, so that cost-utility analysis can be undertaken when health outcomes are only assessed by the OSS score in studies of patients with shoulder pathology. The secondary objective was to develop and assess the performance of different mapping algorithms to estimate the EQ-5D-5L health utility index from OSS responses.

## Methods

### Data

Patient level data were obtained from four randomised controlled trials (RCTs) in the United Kingdom: “Can Shoulder Arthroscopy Work (CSAW)”, “Proximal Fracture of the Humerus: Evaluation by Randomisation (Profher)”, “United Kingdom Rotator Cuff Trial (UKUFF)” and “United Kingdom Frozen Shoulder Trial (UKFROST)” [13–16]. These RCTs evaluated surgical and non-surgical interventions for different types of shoulder problems in different patient cohorts. All trials collected both OSS and EQ-5D responses as primary and secondary outcome measures from patients at specific time-points throughout their follow-up, ranging from baseline to 24 months following intervention (trial details can be found in the Supplementary material).

### Source and target measures

The OSS is a 12-item, unidimensional PROM designed and developed for assessing outcomes following shoulder surgery [12]. A score between zero and four is assigned to each of its 12 items. Items encompass different elements of shoulder pain and the effect of shoulder function on daily activities, for example “Q1: How would you describe the worst pain you had from your shoulder?” and “Q3: Have you had any trouble getting in and out of a car or using public transport because of your shoulder?”. These individual scores are summed to give a total OSS score ranging from zero (worst outcome) to 48 (best outcome). Like the Oxford Hip and Knee scores, the OSS can be treated as a continuous variable under the assumption that it reflects levels of clinical severity [17].

The EQ-5D is a widely used generic measure of health where a number of different health states based on five dimensions (mobility, self-care, usual activities, pain/discomfort, anxiety/depression) can be translated into a summary health index using a preference-based valuation set. Whereas a higher value of the EQ-5D health index represents a better health state, a higher value of each individual domain score represents a poorer health state.

The EQ-5D-3L, the original version of the EQ-5D used, records three levels of responses to each domain (1, 2, 3) and has an established value set for the UK population [18]. The EQ-5D-5L was developed more recently, allowing five levels of responses (1, 2, 3, 4, 5), and a value set for the English population was published in 2018 [19]. However, NICE issued a position statement in 2019 recommending that the health index using 5L data is only calculated following the use of a crosswalk mapping algorithm to 3L first [20, 21]. NICE subsequently recommended further valuation studies for the EQ-5D-5L [22].

Three studies (CSAW, Profher, UKUFF) collected the EQ-5D-3L whilst UKFROST collected the EQ-5D-5L. As the primary objective of this study was focused on the 3L version, the detailed results presented here pertain to pooled data from the CSAW, Profher and UKUFF trials. The same study methodology was applied separately to data from the UKFROST trial; detailed results for those analyses are reported in Supplementary material 2 and 3 where the health index was calculated using the 5L value set directly, and the crosswalk mapping function to 3L, respectively. All analyses were implemented using the *eq5d * package in R.

Like existing mapping studies, we were interested in cross-sectional mapping to estimate health states without necessitating repeated per-patient follow-up observations. Therefore, we pooled all patients’ paired OSS and EQ-5D responses for the studies using the EQ-5D-3L together, giving a total of 4061 (CSAW-939, Profher-750, UKUFF-2372) paired outcome observations. Most patients provided questionnaire responses at more than one time-point; we accounted for this data clustering using the R packages *miceadds* and *estimatr* to produce robust standard errors for the reported model coefficients.

### Models

Two categories of mapping approaches were evaluated: transfer to utility (TTU) regression and response mapping. A summary of these models is shown in Table 1 (details in Sect. 3, Supplementary material 1).

**Table 1:** Summary of models

Models	Description	Covariate(s)	Output
*Time to utility (TTU) regression*			
Univariate linear	Ordinary least squares linear regression	Total OSS score	Predicted EQ-5D health index
Linear splines	Ordinary least squares linear regression with piecewise function introducing knots	Total OSS score	Predicted EQ-5D health index
Polynomial	Squared and cubic polynomial models	Total OSS score	Predicted EQ-5D health index
Cubic splines	Squared and cubic polynomial models with piecewise function introducing knots	Total OSS score	Predicted EQ-5D health index
Multivariable linear	Ordinary least squares linear regression	Question-level OSS score for each of 12 questions	Predicted EQ-5D health index
Two-part	Step 1: logistic regression to identify patients with a probability greater or equal to 0.5 of having a health index of 1. Step 2: ordinary least squares linear regression for patients with a probability less than 0.5 of having a health index of 1 from step 1	Total OSS score	Predicted EQ-5D health index
Tobit	Censored regression model designed for left or right censoring in the dependent variable. Bounds for EQ-5D index used are -0.594 to 1	Total OSS score	Predicted EQ-5D health index
Adjusted limited dependent variable mixture model (ALDVMM)	Tailored model for mapping that replaces the underlying normal distributions with beta distributions	Question-level OSS score for each of 12 questions	Predicted EQ-5D health index
*Response mapping*			
Ordered logistic regression	Ordinal regression model to predict the probability of responses 1, 2 or 3 for each EQ-5D domain	Question-level OSS score for each of 12 questions	Predicted response category (1,2 or 3) for each EQ-5D domain
Seemingly unrelated regression (SUR)	Simultaneous estimation of OLS linear equations to predict each EQ-5D domain response	Question-level OSS score for each of 12 questions	Predicted response category (1,2 or 3) for each EQ-5D domain
*Regularised models (LASSO, ridge and elastic net regression)*			
Multivariable linear	LASSO, ridge regression and elastic net regression regularisation techniques applied to above multivariable model	Question-level OSS score for each of 12 questions	Predicted EQ-5D health index
Ordered logistic regression	LASSO, ridge regression and elastic net regression regularisation techniques applied to above ordered logistic regression model	Question-level OSS score for each of 12 questions	Predicted response category (1,2 or 3) for each EQ-5D domain

*OSS* Oxford Shoulder Score, *LASSO* Least Absolute Shrinkage and Selection Operator

TTU regression approaches aim to use OSS responses to directly predict the EQ-5D health index. We evaluated several different TTU regression models including univariate linear, polynomial, multivariable linear, two-part logistic-linear, tobit and adjusted limited dependent variable mixture models (ALDVMM). We investigated the effect of introducing a piecewise (spline) function to display different coefficients over different ranges of the OSS. From our data, a considerable number of patients (17.9%) reported an EQ-5D health state of “11111” indicating full health (i.e. health utility index equal to 1). The univariate linear regression model would not predict a health index equal to 1, so we developed a two-part model consisting of a logistic and linear regression component. The logistic regression component predicts the probability of a patient to have an EQ-5D health index of 1, and the linear component predicts the health state of the remaining patients. Tobit models allow for a linear relationship between the OSS and EQ-5D with censoring of values at the lower and upper bounds of possible EQ-5D values. ALDVMM is a tailored model developed specifically for mapping that replaces the underlying normal distributions with beta distributions that can be used for bounded outcomes [23]. Sequential likelihood-ratio tests were used to compare nested multivariable models when reducing the number of covariates to improve model parsimony.

The aim of response mapping is to predict responses to the EQ-5D questions rather than to directly predict the health index [24]. The EQ-5D health index can then be calculated using country-specific tariffs. Interest in response mapping has grown due to certain limitations with TTU regression approaches [25]. Firstly, the distribution of health utilities is often not linear and there is a significant mass of observations at the upper boundary of one. This means that regression techniques may not be able to capture the true association between a predictor and the health index directly. Second, TTU regression models for EQ-5D are country-specific and thus less generalisable, due to requiring specific tariffs to convert health states to health utility. However, response mapping approaches require more granular data and are often more computationally intensive.

We used ordered logistic regression and seemingly unrelated regression (SUR) models. The ordered logistic regression model used all 12 OSS question responses to predict the response categories (1, 2 or 3) for each EQ-5D domain. The health index was subsequently calculated using the UK tariff from the predicted EQ-5D-3L questionnaire responses. SUR accounted for the potential correlations between elements of the equations for each EQ-5D domain.

We evaluated the effect of the addition of age and sex as predictor variables to each model. Regularisation techniques such as LASSO (Least Absolute Shrinkage and Selection Operator), ridge and elastic net regression reduce the risk of overfitting by reducing parameters and shrinking a model. We implemented these techniques on the multivariable linear and ordered logistic regression models and their effect on model performance was evaluated.

### Validation

We did not have access to a dedicated validation dataset, but our sample size was sufficiently large to split it randomly into training and testing samples. Patients were randomly assigned into either the training or testing sample with a 70:30 split, respectively. Models were developed using the training sample. All models were first evaluated through internal validation where the model was fit to the training sample. We then examined model fit on the testing sample. Given the potential for this random split of our dataset to not be truly random, we carried out a 100-fold repeated random split of the training and testing samples. Each time, models were developed on a different training sample and their performance evaluated on a different testing sample. We reported the overall model performance across repeated testing samples. All available data were subsequently used to calculate the final model parameters reported in this study.

The developed models’ performance was then evaluated against subsets of the original testing sample, where each subset consisted of data from just one trial at a time, to evaluate model performance against known heterogeneity.

### Model performance

Our primary measures of model performance were the mean absolute error (MAE) and mean square error (MSE) between the observed and predicted EQ-5D health index scores. We were primarily interested in overall model performance on the testing sample averaged across the 100-fold repeated random split.

Other performance metrics are also important as they reflect different aspect of prediction accuracy. We assessed the deviation of the predicted mean from the observed mean health index and estimated the linear correlation between observed and predicted health index scores. We reported model calibration by examining how model performance varied depending on different tenths of the predicted health index and of the observed total OSS score.

We followed the ‘MAPS (MApping onto Preference-based measures reporting Standards) reporting statement’ and ‘ISPOR Mapping to Estimate Health-State Utility Values from Non–Preference-Based Outcomes Measures for Cost per QALY Economic Analysis Good Practices Task Force Report’ when reporting this mapping study [26, 27]. All statistical analysis were undertaken using R software [28].

## Results

### Data

For the EQ-5D-3L analysis, there were missing data in a total of 356 outcome observations (8.8% of the initial dataset), so the final dataset consisted of 3705 paired outcomes observations from 1202 patients, including patient age and sex. The training sample consisted of 2586 observations from 841 patients. The testing sample consisted of 1119 observations from 361 patients. Missing data analysis is shown in Sect. 2, Supplementary material 1.

**Fig. 1:**
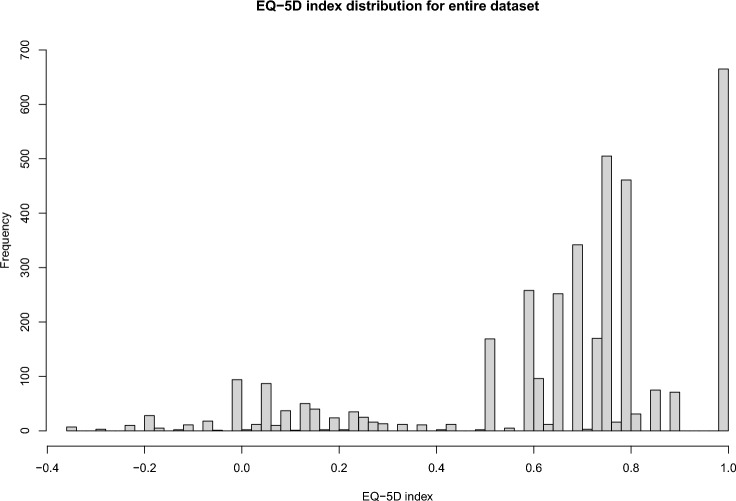
EQ-5D health index distribution for the entire dataset

**Fig. 2:**
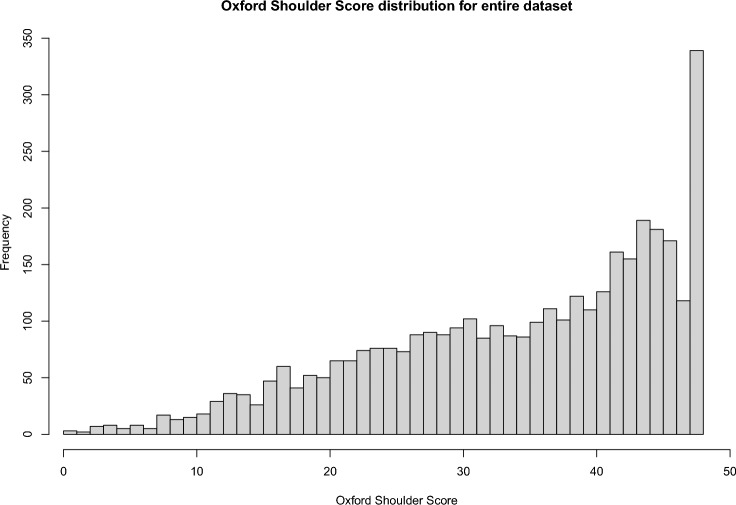
Oxford Shoulder Score distribution for the entire dataset

Figures 1 and 2 show the distribution of the EQ-5D health index and OSS score for the complete dataset. The EQ-5D health index distribution demonstrates a gap in values towards the upper end as expected in accordance with the UK EQ-5D tariff. At baseline, the OSS scores were approximately normally distributed, but over time they appeared increasingly negatively skewed with a greater proportion of observations at higher values (Sect. 1, Supplementary material 1).

**Fig. 3:**
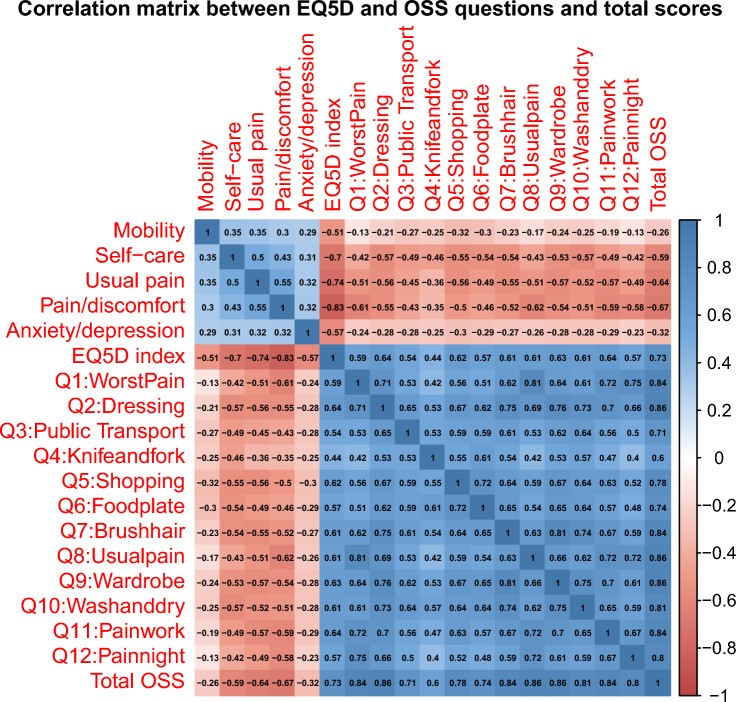
Correlation matrix between EQ-5D and Oxford Shoulder Score questions and total scores. Spearman correlation coefficient values shown in colour-coded text depending on the magnitude according to the colour gradient scale on the right-hand side. *OSS* Oxford Shoulder Score

Figure 3 shows the correlation matrix between all OSS and EQ-5D questions. This confirmed a positive correlation of 0.73 between EQ-5D and OSS. Within each instrument there was a positive correlation between all questions. Within the OSS, questions Q2: “Have you had any trouble dressing yourself because of your shoulder?” and Q8: “How would you describe the pain you usually had from your shoulder?” and Q9: “Could you hang your clothes up in a wardrobe, using the affected arm? (whichever you tend to use)” had the highest correlation (0.86) with the total OSS score, while question Q4: “Have you been able to use a knife and fork at the same time?” had the lowest correlation (0.60). Within the EQ-5D, the pain/discomfort domain had the strongest negative correlation (− 0.83) with the EQ-5D health index while the mobility domain had the weakest negative correlation (− 0.51).

### Models

**Fig. 4:**
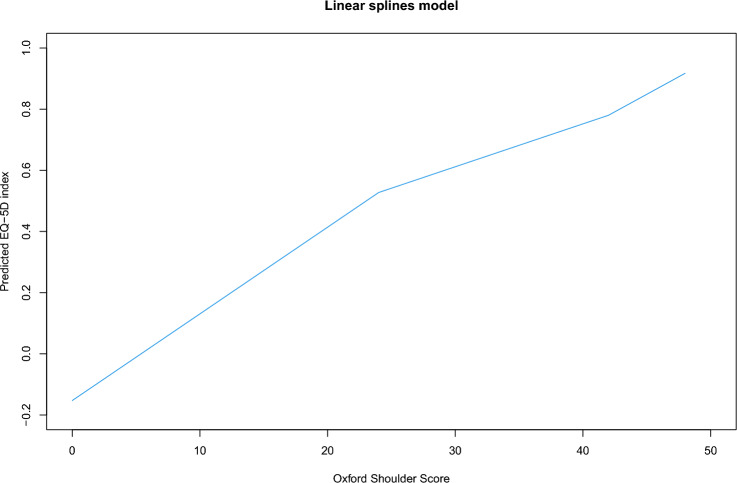
Linear splines model predicting EQ-5D from total Oxford Shoulder Score consisting of two knots. Knots at OSS = 24 and OSS = 41. Oxford Shoulder Score (OSS). Enter the OSS score of the patient in one of the three following equations depending on the range within which the score lies. The equation will output the predicted EQ-5D index as corresponds to the linear splines model graph. Predicted EQ-5D index = − 0.13757 + 0.0276*OSS score (valid for 0 ≤ OSS ≤ 24), Predicted EQ-5D index = 0.19563 + 0.0137*OSS score (valid for 24 < OSS ≤ 41), Predicted EQ-5D index = − 0.18867 + 0.0231*OSS score (valid for 41 < OSS ≤ 48)

The univariate linear model using the total OSS score as a single covariate to predict the EQ-5D health index was the simplest model and was statistically significant. For the linear splines model, knot locations at OSS values of 24 and 41 gave the best model performance, with different equations derived for each linear segment. The gradients of the first (OSS ≤ 24) and last (OSS > 41) linear segments were greater than that of the middle section (Fig. 4).

Two polynomial models were fit, but the cubic model had significantly better model fit compared to the squared model following a likelihood-ratio test. A single knot at an OSS value of 22 was used for the cubic spline model; adding further knots did not improve model performance.

For the multivariable linear model, sequential likelihood-ratio testing resulted in dropping two OSS questions (Q7 and Q9). Question 11: “How much has the pain from your shoulder interfered with your usual work (including housework)?” had the largest coefficient (0.04235) while question 12: “Have you been troubled by pain from your shoulder in bed at night?” had the smallest coefficient (0.01237).

For the ordered logistic regression response mapping model, all five individual domain models were statistically significant but certain covariates within each model were not. Removing these covariates led to reduced model performance on the testing sample, so all 12 OSS questions were retained. There was considerable variation in the pattern of OSS question coefficients between the five different EQ-5D domains. However, certain patterns made intuitive sense, for instance: Question 1 “How would you describe the worst pain you had from your shoulder?” had the greatest (negative) coefficient for the pain/discomfort domain and question 5 “Could you do household shopping on your own?” had the greatest (negative) coefficient for the mobility domain.

For both response mapping approaches, the coefficients of certain covariates were unexpectedly positive, and some were concurrently significant. This was probably due to a modest degree of collinearity between the 12 OSS questions. Removing certain non-significant predictor variables reduced the model’s predictive performance and did not resolve the unexpected sign issue.

Adding age and sex as covariates appeared to make very little difference in the models’ predictive ability, with ALDVMM performing worse. Among ridge, LASSO and elastic net regression, LASSO regression produced regularised multivariable and ordered logistic regression models with the best predictive ability. However, the improvement in predictive ability on the testing sample was minor for the multivariable model, and performance was worse for the ordered logistic model.

Details for model parameters and model performance are reported in Sects. 4 and 5 of the Supplementary material, respectively.

### Validation

In internal validation, the univariate linear, cubic, splines and multivariable linear models’ predicted mean EQ-5D health index deviated the least (10^–15^) from the observed mean while the SUR model’s predicted mean deviated the most (0.06531). The multivariable linear model had the lowest MSE (0.0420) while the SUR model had the lowest MAE (0.136). All models performed better at OSS values greater than the median for the cohort (Table 5.1, Supplementary material 1).

Model performance was subsequently evaluated against the testing sample, and we reported average performance metrics across the 100-fold repeated random split (Table 2). Predicted mean EQ-5D health index values deviated no more than 0.0647 from the observed mean, with the cubic splines model deviating the least (0.00304) and the response mapping models deviating the most (ordered logistic: 0.0647, SUR: 0.0612). The multivariable model had the lowest MSE (0.04148), followed closely by the linear splines (MSE 0.04151) and cubic splines models (MSE 0.04151). The response mapping models had the greatest MSE (0.0524). The response mapping models had the lowest MAE (ordered logistic: 0.137, SUR: 0.136) whereas the two-part model had the greatest MAE (0.156). All models performed better at OSS values greater than the median for the tested cohort. Model performance was consistent across the 100-fold repeated splits as demonstrated by the standard deviations of the performance metrics.

**Table 2:** Summary model performance indicators on testing sample validation. Mean and standard deviation (sd) for each performance measure following 100-fold random splits into training and testing samples. Results are based on model performance on the testing samples

Model	Mean fitted health index		Difference of means (observed-predicted)		Mean square error (MSE)						Mean absolute error (MAE)						
					Total		OSS < median		OSS ≥ median		Total		OSS < median		OSS ≥ median		
	Mean	sd	Mean	sd	Mean	sd	Mean	sd	Mean	sd	Mean	sd	Mean	sd	Mean		sd
Univariate linear	0.664638	0.00671	0.003242	0.010342	0.042018	0.00263	0.058294	0.003418	0.026637	0.003712	0.153767	0.003612	0.196681	0.006388	0.113221		0.004441
Linear splines	0.664819	0.006894	0.003061	0.010137	0.04151	0.002707	0.058294	0.003418	0.026637	0.003712	0.148607	0.00365	0.196681	0.006388	0.113221		0.004441
Polynomial (cubic)	0.664745	0.006825	0.003135	0.010222	0.04151	0.002684	0.058294	0.003418	0.026637	0.003712	0.149894	0.003601	0.196681	0.006388	0.113221		0.004441
Cubic splines	0.664842	0.006942	0.003038	0.010274	0.041637	0.003411	0.058294	0.003418	0.026637	0.003712	0.148123	0.003877	0.196681	0.006388	0.113221		0.004441
Multivariable linear	0.664454	0.006554	0.003426	0.010007	0.041475	0.002586	0.058294	0.003418	0.026637	0.003712	0.152137	0.003649	0.196681	0.006388	0.113221		0.004441
Two-part	0.657198	0.007485	0.010682	0.011105	0.044989	0.002552	0.060155	0.003435	0.03066	0.003597	0.156177	0.003805	0.205348	0.006072	0.109709		0.004882
Tobit	0.695022	0.007896	− 0.02714	0.010554	0.043843	0.003025	0.057795	0.003422	0.030658	0.004675	0.151928	0.004136	0.19163	0.006559	0.114414		0.006119
ALDVMM	0.683496	0.013547	− 0.01562	0.017098	0.048158	0.005329	0.068817	0.00912	0.028657	0.003675	0.15572	0.006586	0.192058	0.00999	0.121407		0.007165
Ordered logistic	0.729761	0.008574	− 0.06467	0.008817	0.052371	0.00391	0.073326	0.006417	0.032549	0.004371	0.137165	0.005738	0.171474	0.00961	0.104715		0.006288
SUR	0.729091	0.00783	− 0.06121	0.009548	0.051197	0.004087	0.071539	0.006499	0.031986	0.004411	0.136057	0.005922	0.170404	0.009864	0.103615		0.006069

*OSS* Oxford Shoulder Score, *ALDVMM* Adjusted Limited Dependent Variable Mixture Model, *SUR* Seemingly Unrelated Regression

Plots of the residuals (observed – predicted health index) versus the observed EQ-5D health index values are shown in Sect. 5, Supplementary material 1. For all models, the predicted health index was underestimated at better observed health states and overestimated at poorer observed health states.

The patterns of model performance across different ranges of predicted EQ-5D health index or different ranges of observed OSS score were similar. Most models performed better in the middle of the range of health states, with ALDVMM having the most notable such pattern (see Supplementary material). Response mapping performed best in both extremes of health states while the multivariable linear model performed best in average health states.

The developed models were tested against subsets of the testing sample consisting of a different trial at a time, with models performing better on the Profher subset which had notably higher perfect-health state respondents (Sect. 5, Supplementary material 1).

From our secondary analysis using EQ-5D-5L data, the multivariable linear and cubic splines models had the lowest MSE values in the 5L value set and 3L crosswalk analyses, respectively. The SUR model had the lowest MAE in both analyses. Performance metrics were lower in the 5L value set models (MSE 0.024–0.030, MAE 0.105–0.114) compared to the 3L crosswalk. Detailed results can be found in Supplementary material 2 (5L value set) and Supplementary material 3 (3L crosswalk).

## Discussion

In this study we demonstrated that the OSS can be accurately mapped to the EQ-5D using both TTU regression and response mapping approaches. We primarily focused on EQ-5D-3L but also undertook a separate analysis on EQ-5D-5L data using data from a single trial.

### EQ-5D-3L

All models evaluated performed well and were able to predict the EQ-5D health index with high accuracy. The overall model predictive ability (MSE range: 0.0415–0.0524, MAE range: 0.136–0.156) was in keeping with other mapping studies that report an RSME around 0.20 (MSE ~ 0.04) and MAE around 0.15 [29–32]. While MSE and MAE are two key performance metrics, comparative model performance was not always consistent in both metrics, likely due to the different optimisation methods employed by different models.

All models demonstrated variation in performance across the range of the observed OSS and predicted EQ-5D health index. Generally, predictive performance was greatest in the mid-range and best health states, and lowest in poor health states. The tendency for models to perform in this way when mapping condition-specific to generic outcome measures has been previously identified [29, 31–33]. However, it is important to note that the number of observations in our study population with poor health were considerably fewer than those with good health; consequently, the model parameters will be skewed to improve prediction for good health states. From the plots of the residuals versus the observed EQ-5D health index it was apparent that all models overpredict poor health states and underpredict the best health states, as has been previously reported [34].

Based on the results of the univariate linear model, 48.6% of the variation in the EQ-5D health index in our dataset could be explained by the variation in the OSS score. While there is no published literature on the OSS score available to compare this against, studies mapping the Oxford Hip Score and Oxford Knee Score onto EQ-5D had similar findings (42–69%) [31, 34]. The correlation matrix demonstrates that the Mobility and Anxiety/Depression domains of the EQ-5D had the weakest association with the OSS score (Fig. 3). This is also reflected in the covariates’ significance levels in the ordered logistic model. This could be expected given that the shoulder is not as critical for overall mobility as are lower limb joints such as the hip and knee. Furthermore, there is no question in the OSS questionnaire that directly asks about mental health elements such as anxiety and depression. These factors reduce the ability of any mapping approach to accurately predict the EQ-5D health index from the OSS score, a problem that has also been encountered with other musculoskeletal disease-specific outcome measures [31, 34, 35].

When choosing a model in future mapping exercises, investigators will need to take several factors into consideration. Certain models such as the univariate linear and linear splines models are simpler and do not require dedicated statistical software to implement. When considering both MSE and MAE metrics, the linear splines model performed best overall while offering a user-friendly application, and we can recommend this model for general use (Fig. 4). Although comparatively less accurate over poorer and mid-range health states, SUR may offer improved predictive accuracy at perfect or near-perfect-health states. Finally, the timing of questionnaire responses is often closely associated to health state – preoperative responses are likely to be representative of poorer health states when compared to postoperative responses.

### EQ-5D-5L

Model performance using the 5L value set or the 3L crosswalk was similar: splines, multivariable linear and SUR models performed best. Model performance using the 5L value set was slightly better, though it is difficult to ascertain how much of this is due to the added uncertainty from introducing the 3L crosswalk algorithm itself. Calibration performance was similar to EQ-5D-3L models, and the improved model performance at the best health states was more marked. The model performance metrics were better than the EQ-5D-3L models, though this comparison may not be meaningful given that the 5L models were developed and validated only on data from patients with frozen shoulder, making the models less generalisable.

This study is not without limitations. We were unable to externally validate these models but carried out a 100-fold repeated random split of the training and testing samples and reported average performance metrics across splits. We were faced with the option to retain one trial as a designated validation dataset, but this would have come at the expense of model generalisability given the broad shoulder conditions represented by participants from multiple surgical shoulder trials. However, we examined the developed models’ performance on trial-specific subsets of the testing sample. As expected, this highlighted some differences between trials (Profher had a greater proportion of perfect-health responders in terms of OSS score), but overall, largely consistent comparative model performance. While all models performed very well in mid-range and better health states, researchers need to be aware that model performance may be reduced in the lower three tenths of OSS score (OSS 0–15). Formal external validation of the developed models remains to be done.

## Conclusion

The OSS is now commonly used throughout the world to monitor surgical outcomes. It is used by several international shoulder replacement registries that are becoming increasingly important in guiding contemporary practice. The mapping models developed and evaluated in this study will enable researchers to obtain accurate and reliable estimates of the EQ-5D when only the OSS score is available, helping to address the paucity of cost-utility analysis. TTU regression models will facilitate direct estimation of the EQ-5D health index, while response mapping models allow tariffs from different countries to be used. Although several factors need to be considered in choosing the best model for a particular application, the linear splines model performed well, and its straight-forward implementation can facilitate widespread uptake. If these mapping algorithms are used to inform health economic evaluations, it is necessary to incorporate the uncertainty generated by the mapping process into any analysis.

### Supplementary Information

Below is the link to the electronic supplementary material.

Supplementary file1 (DOCX 5695 KB)

Supplementary file2 (DOCX 3106 KB)

Supplementary file3 (DOCX 4827 KB)

Supplementary file4 (XLSX 84 KB)
