# Supplementary material for: Mapping the Oxford Shoulder Score onto the EQ-5D utility index
Source: Qual Life Res. 2022 Sep 28;32(2):507–18. doi: 10.1007/s11136-022-03262-4 (PMC9911508; doi:10.1007/s11136-022-03262-4)
Supplement: Supplementary file 3 — Supplementary file3 (DOCX 4827 KB) [file 11136_2022_3262_MOESM3_ESM.docx]

**Supplementary material 3: EQ-5D-5L(3L Crosswalk)**

Table of Contents

[Article title 3](#_Toc113786532)

[Journal name 3](#_Toc113786533)

[Authors 3](#_Toc113786534)

[Section 1: Trials and datasets 5](#_Toc113786535)

[Trial details 5](#_Toc113786536)

[Gender distributions 5](#_Toc113786537)

[Age distributions 5](#_Toc113786538)

[Comparing EQ-5D-5L and crosswalk value sets 6](#_Toc113786539)

[EQ-5D health index and OSS score distributions 7](#_Toc113786540)

[EQ-5D health index and OSS score distributions per timepoints (in months) of follow-up 7](#_Toc113786541)

[Section 2: Missing data analysis 8](#_Toc113786542)

[Descriptive analysis 8](#_Toc113786543)

[UKFROST 8](#_Toc113786544)

[Missing data proportions and combinations 9](#_Toc113786545)

[Missingness per EQ-5D index 10](#_Toc113786546)

[Missingness per OSS score 10](#_Toc113786547)

[Missingness relationships between EQ-5D and OSS 11](#_Toc113786548)

[Completely missing questionnaire responses 13](#_Toc113786549)

[EQ-5D index 13](#_Toc113786550)

[OSS score 14](#_Toc113786551)

[Section 3: Description of models 15](#_Toc113786552)

[TTU regression models 15](#_Toc113786553)

[Univariate linear 15](#_Toc113786554)

[Linear splines 15](#_Toc113786555)

[Polynomial 15](#_Toc113786556)

[Cubic splines 15](#_Toc113786557)

[Multivariable linear 16](#_Toc113786558)

[Two-part 16](#_Toc113786559)

[Tobit 16](#_Toc113786560)

[Adjusted Limited Dependent Variable Mixture Model (ALDVMM) 16](#_Toc113786561)

[Response mapping models 17](#_Toc113786562)

[Ordered logistic model 17](#_Toc113786563)

[Seemingly unrelated regression (SUR) 17](#_Toc113786564)

[Section 4: Model specifications 18](#_Toc113786565)

[Univariate linear 18](#_Toc113786566)

[Linear splines 18](#_Toc113786567)

[Polynomial (cubic) 20](#_Toc113786568)

[Polynomial (squared) 20](#_Toc113786569)

[Cubic splines 20](#_Toc113786570)

[Multivariable linear 22](#_Toc113786571)

[Two-part 23](#_Toc113786572)

[Tobit 23](#_Toc113786573)

[ALDVMM 24](#_Toc113786574)

[Ordered logistic regression 25](#_Toc113786575)

[SUR 26](#_Toc113786576)

[Section 5: Model performance 29](#_Toc113786577)

[Internal validation 29](#_Toc113786578)

[100-fold repeated random splitting of the training and testing samples 30](#_Toc113786579)

[Model residuals 31](#_Toc113786580)

[Calibration plots- performance across tenths 32](#_Toc113786581)

[Section 6: References 34](#_Toc113786582)

# Article title

Mapping the Oxford Shoulder Score onto the EQ-5D utility index

# Journal name

Quality of Life Research

# Authors

Epaminondas M Valsamis, MB BChir, MA(Cantab)

Nuffield Department of Orthopaedics, Rheumatology and Musculoskeletal Sciences,

University of Oxford, Botnar Research Centre, Oxford OX3 7LD,

United Kingdom

David Beard DPhil(Oxon)

Nuffield Department of Orthopaedics, Rheumatology and Musculoskeletal Sciences,

University of Oxford, Botnar Research Centre, Oxford OX3 7LD,

United Kingdom

Andrew Carr DSc

Nuffield Department of Orthopaedics, Rheumatology and Musculoskeletal Sciences,

University of Oxford, Botnar Research Centre, Oxford OX3 7LD,

United Kingdom

Gary S Collins PhD

Centre for Statistics in Medicine, Nuffield Department of Orthopaedics, Rheumatology and Musculoskeletal Sciences, University of Oxford, Oxford OX3 7LD,

United Kingdom

Stephen Brealey PhD

York Trials Unit, Department of Health Sciences, University of York, York, YO10 5DD,

United Kingdom

Amar Rangan FRCS(Tr&Orth)

York Trials Unit, Department of Health Sciences, University of York, York YO10 5DD,

United Kingdom

Rita Santos PhD

Centre for Health Economics, University of York, York, YO10 5DD,

United Kingdom

Belen Corbacho MA

York Trials Unit, Department of Health Sciences, University of York, York YO10 5DD,

United Kingdom

**Jonathan L Rees FRCS(Tr&Orth)

Nuffield Department of Orthopaedics, Rheumatology and Musculoskeletal Sciences,

University of Oxford, Botnar Research Centre, Oxford OX3 7LD,

United Kingdom

**Rafael Pinedo-Villanueva PhD

Nuffield Department of Orthopaedics, Rheumatology and Musculoskeletal Sciences,

University of Oxford, Botnar Research Centre, Oxford OX3 7LD,

United Kingdom

**Co-senior authors

**Corresponding author:**

Epaminondas Markos Valsamis

Nuffield Department of Orthopaedics, Rheumatology and Musculoskeletal Sciences,

University of Oxford, Botnar Research Centre, Oxford OX3 7LD,

United Kingdom

+44 1865 227374

[markos.valsamis@ndorms.ox.ac.uk](mailto:markos.valsamis@ndorms.ox.ac.uk)

# Section 1: Trials and datasets

## Trial details

Table 1.1: Trial details

|  | **UKFROST** |
| --- | --- |
| **Dates** | April 2015- December 2017 |
| **Shoulder problem** | Frozen shoulder |
| **Inclusion criteria** | ≥18 years; clinical diagnosis of unilateral frozen shoulder |
| **Number of participants recruited** | 503 |
| **Treatment groups** | Manipulation under anaesthesia; arthroscopic capsular release; early structure physiotherapy |
| **Paired EQ-5D & OSS questionnaire response follow-up** | Baseline; 3 months; 6 months; 12 months |
| **Number of complete responses per timepoint** | 0 months: 483  3 months: 425  6 months: 398  12 months: 426 |
| **EQ-5D version collected** | 5L |

## Gender distributions

Table 1.2: Gender distributions for UKFROST

|  | UKFROST |
| --- | --- |
| Male | 630 |
| Female | 1102 |
| Total | 1732 |

## Age distributions

Table 1.3: Age distributions for UKFROST

|  | UKFROST |
| --- | --- |
| **Age** (mean(SD)) | 54.51(7.56) |

## Comparing EQ-5D-5L and crosswalk value sets

This Supplementary material focuses on the health index values derived using the 5L to 3L crosswalk algorithm and subsequently obtaining health index values (van Hout et al., 2012). The graph below demonstrates the difference in the deduced health index values when compared to deriving the health index values directly from the EQ-5D-5L responses using the value set published by Devlin and colleagues which we describe in Supplementary material 3 (Devlin et al., 2018).


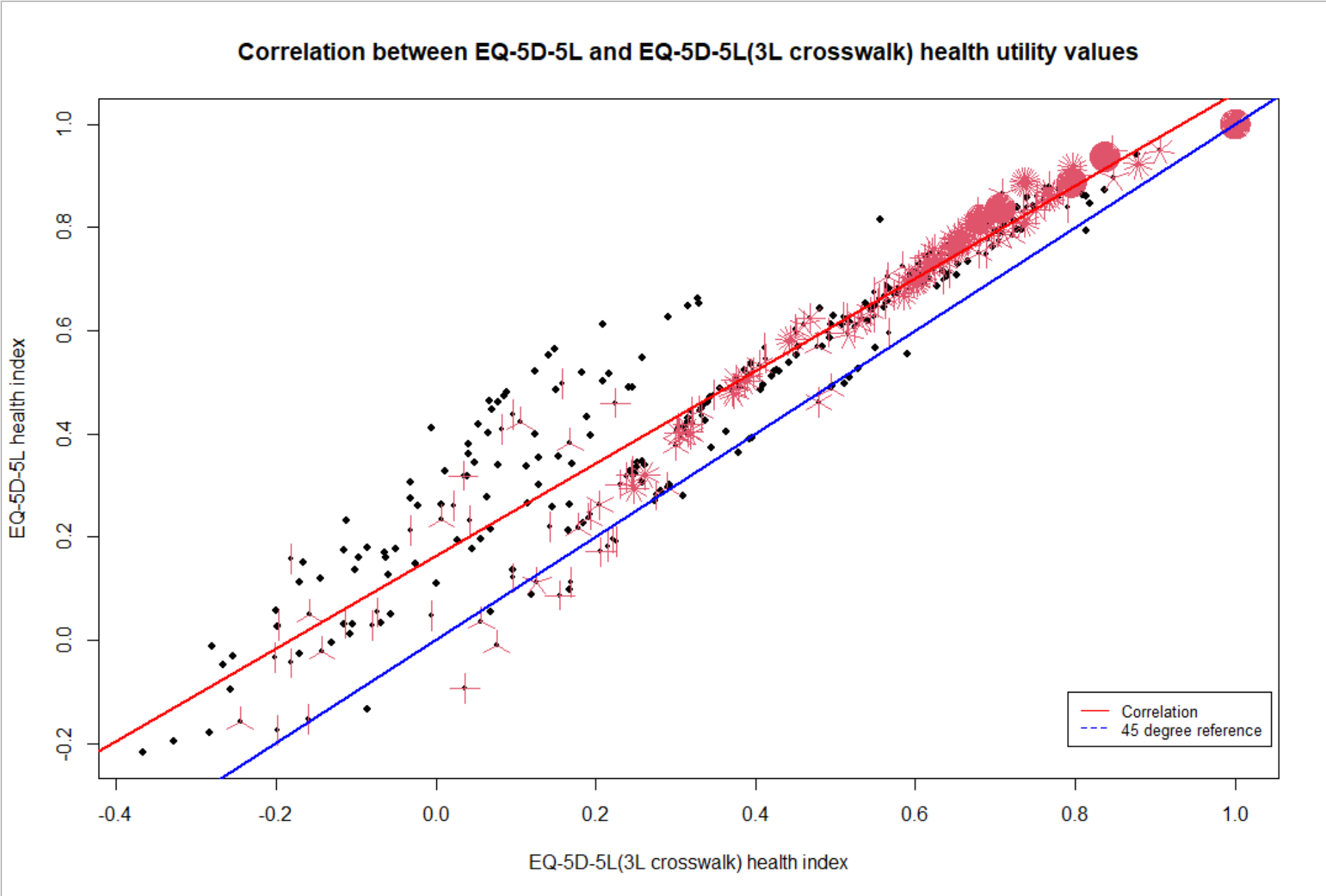


## EQ-5D health index and OSS score distributions


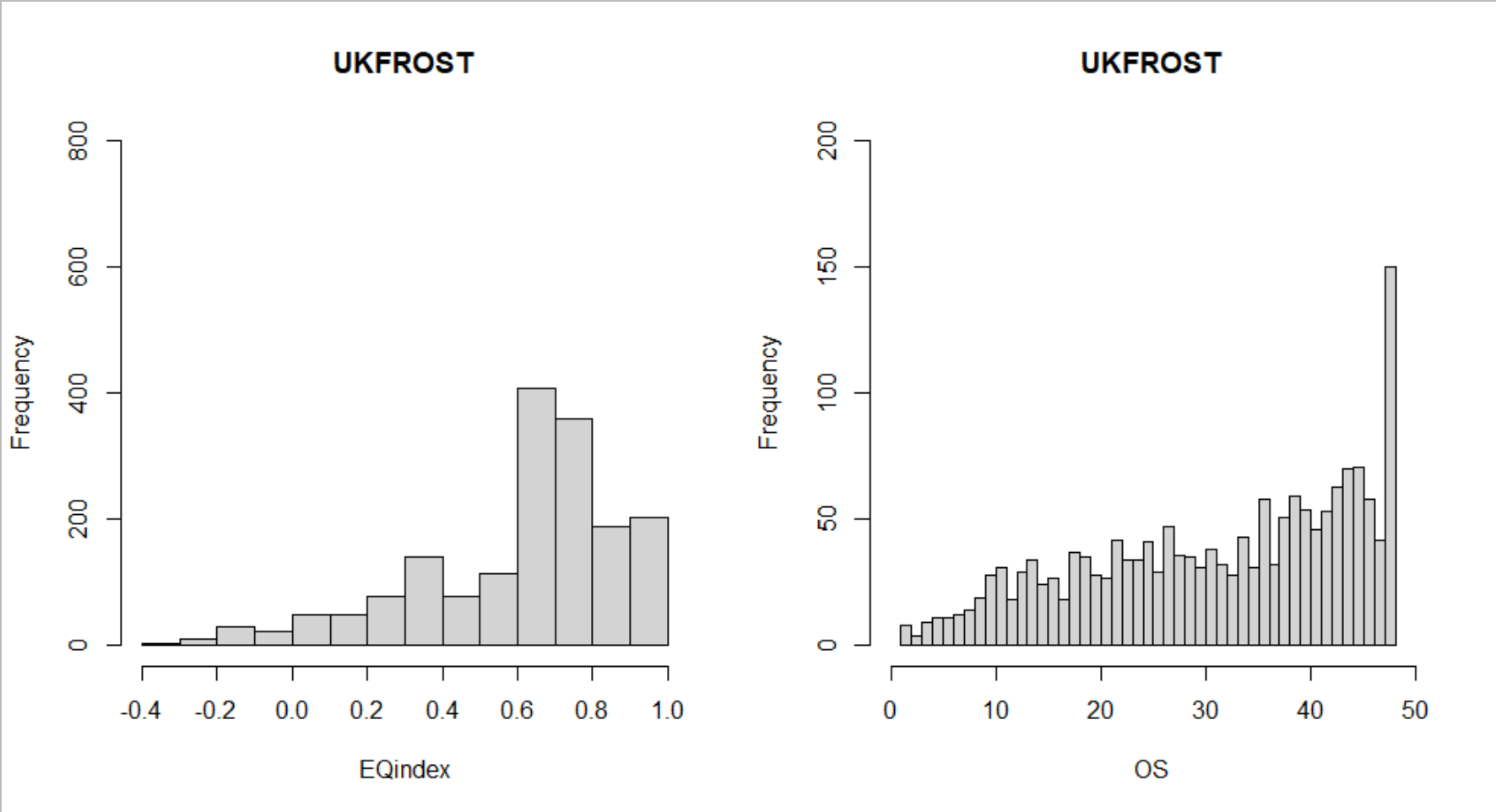


## EQ-5D health index and OSS score distributions per timepoints (in months) of follow-up


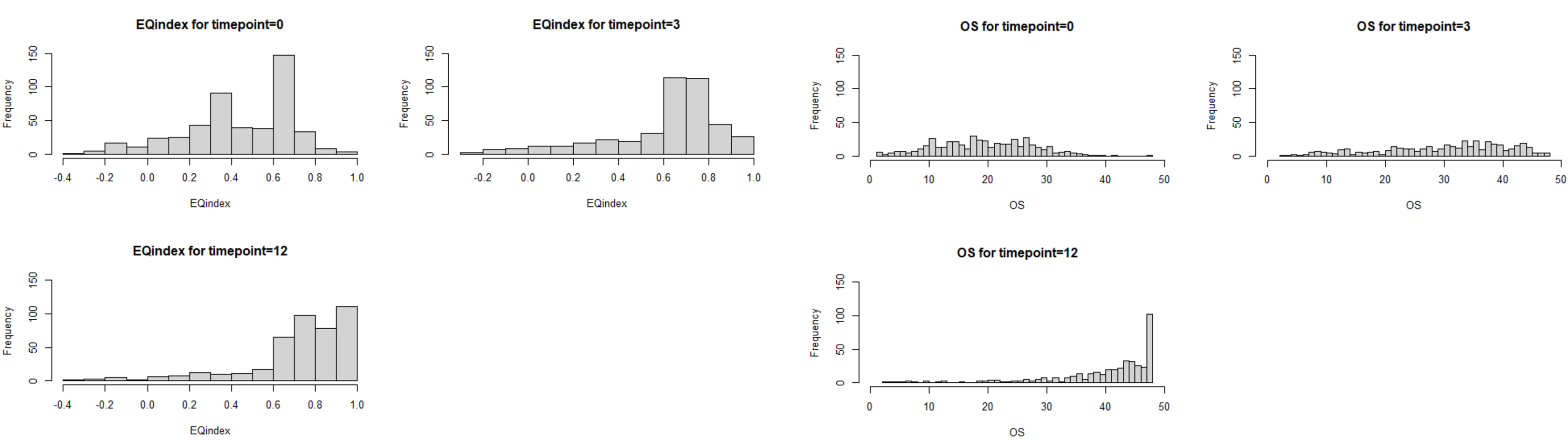


# Section 2: Missing data analysis

## Descriptive analysis

There were missing data in a total of 280 outcome observations (13.9% of the initial dataset). On investigating missing data further, 184 (65.7%) of all missing entries had completely missing data, i.e. no EQ-5D or OSS score responses at all, indicating patients had not returned their questionnaire for a particular time-point. As no patients had a completely missing questionnaire response at baseline, we compared the EQ-5D health index and OSS scores of patients that had subsequent missing responses with those from patients that always responded. There was no statistically significant difference in the EQ-5D health index, but a small, statistically significant increase in the mean OSS score (difference=4.2 less in those who always responded).

There was no apparent association between the missingness of data with patients’ health as measured by either EQ-5D or the OS score. We therefore decided to perform complete-case analysis to avoid the additional hurdles and uncertainty associated with imputation methods in the development of a mapping algorithm ^1^.

More granular missing data information are shown below.

### UKFROST

n=2012

86.08% present (1732)

13.92% missing (280)

OF WHICH

65.6% NO EQ-5D or OSS questions at all (#184 – 9.15%)

7.5% All OSS but only UA from EQ-5D (#21 – 1.04%)

5% NO UA (#14 – 0.70%)

3.6% NO UA or any OSS question response (#10 – 0.50%)

## Missing data proportions and combinations

The bar plot on the left shows the proportion of missing values present in each variable from most to least missing. The diagram on the right shows the combinations of missing variables, from top to bottom, in order of increasing frequency; red indicates missing, blue indicates present.


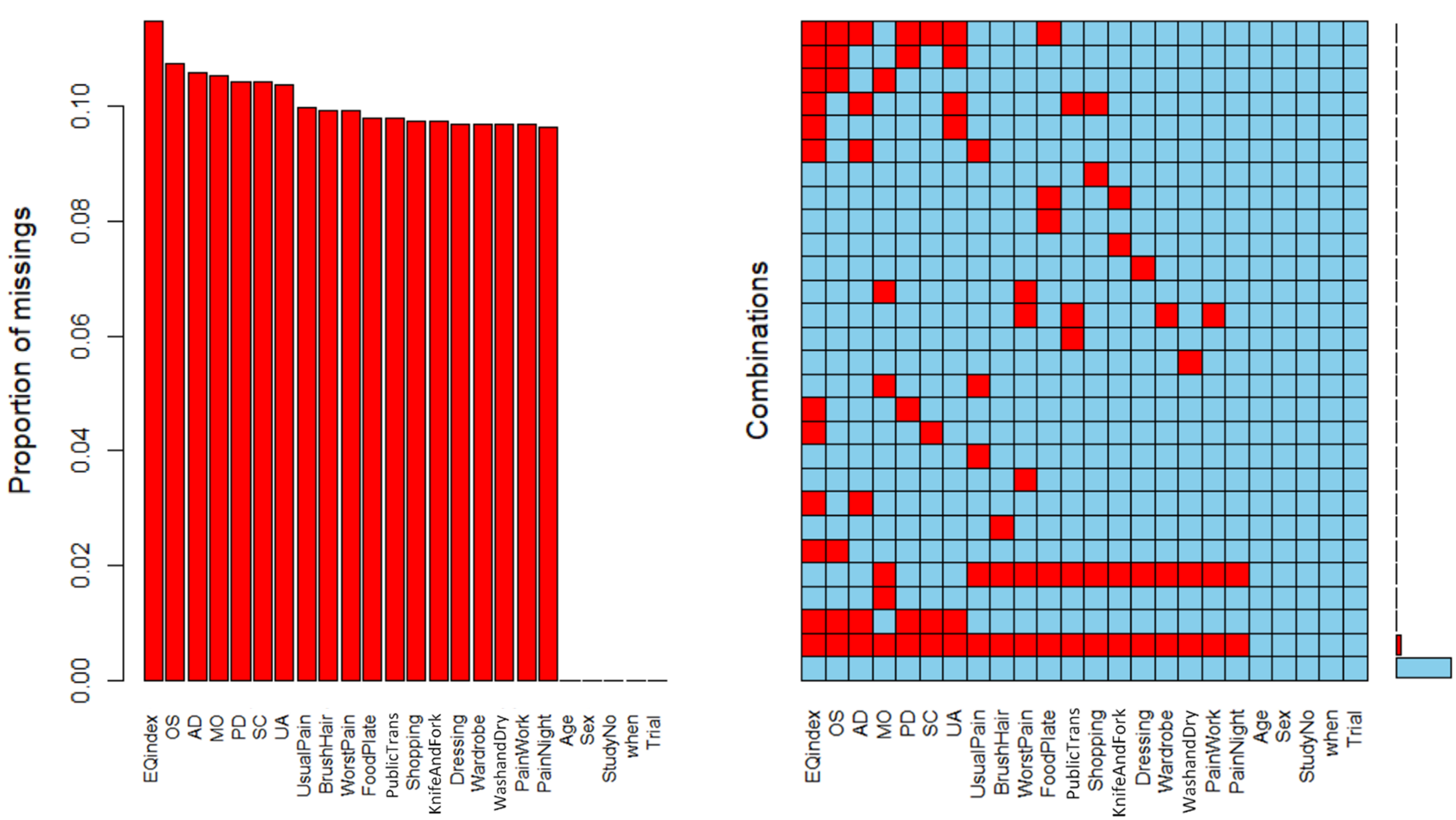


### Missingness per EQ-5D index

Missing in red. Low values in white, high values in black.


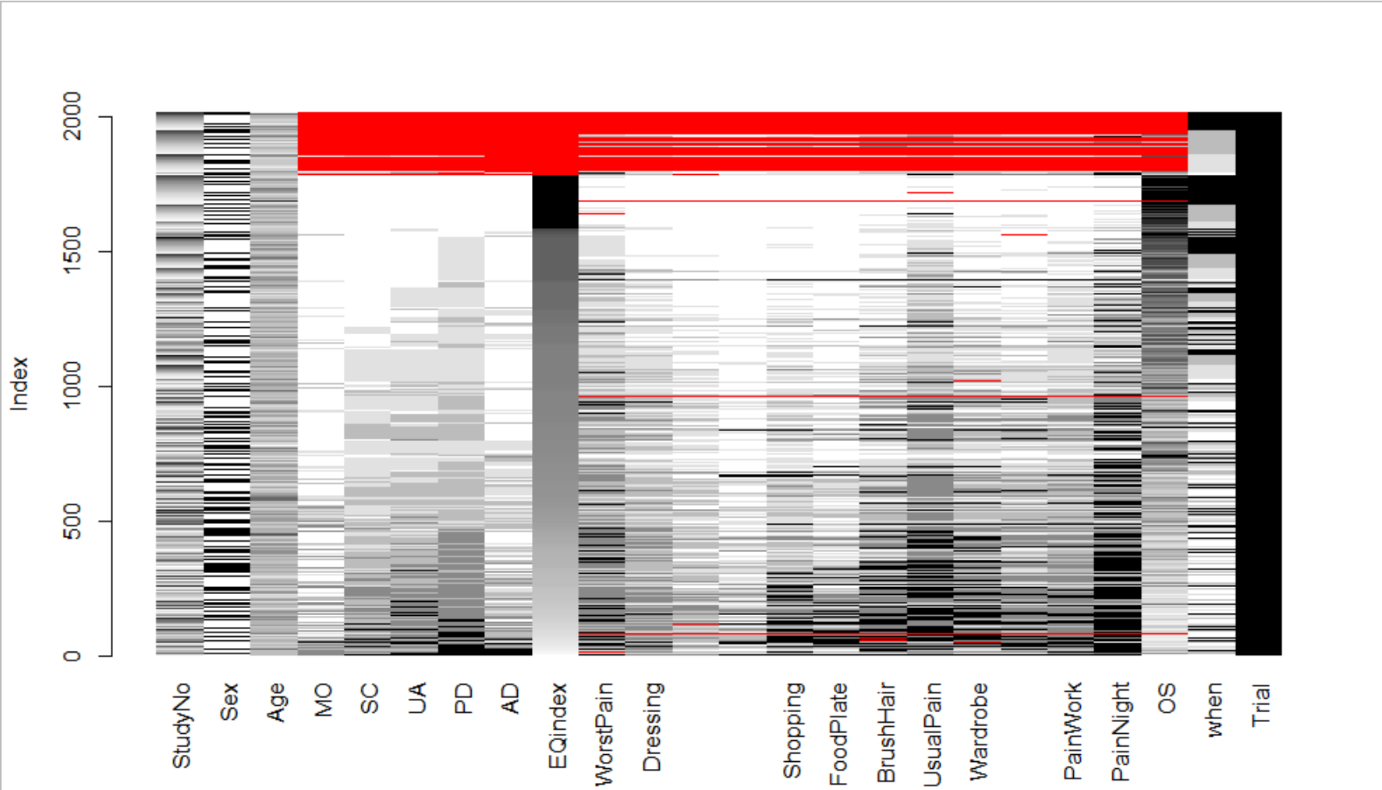


### Missingness per OSS score

Missing in red. Low values in white, high values in black.


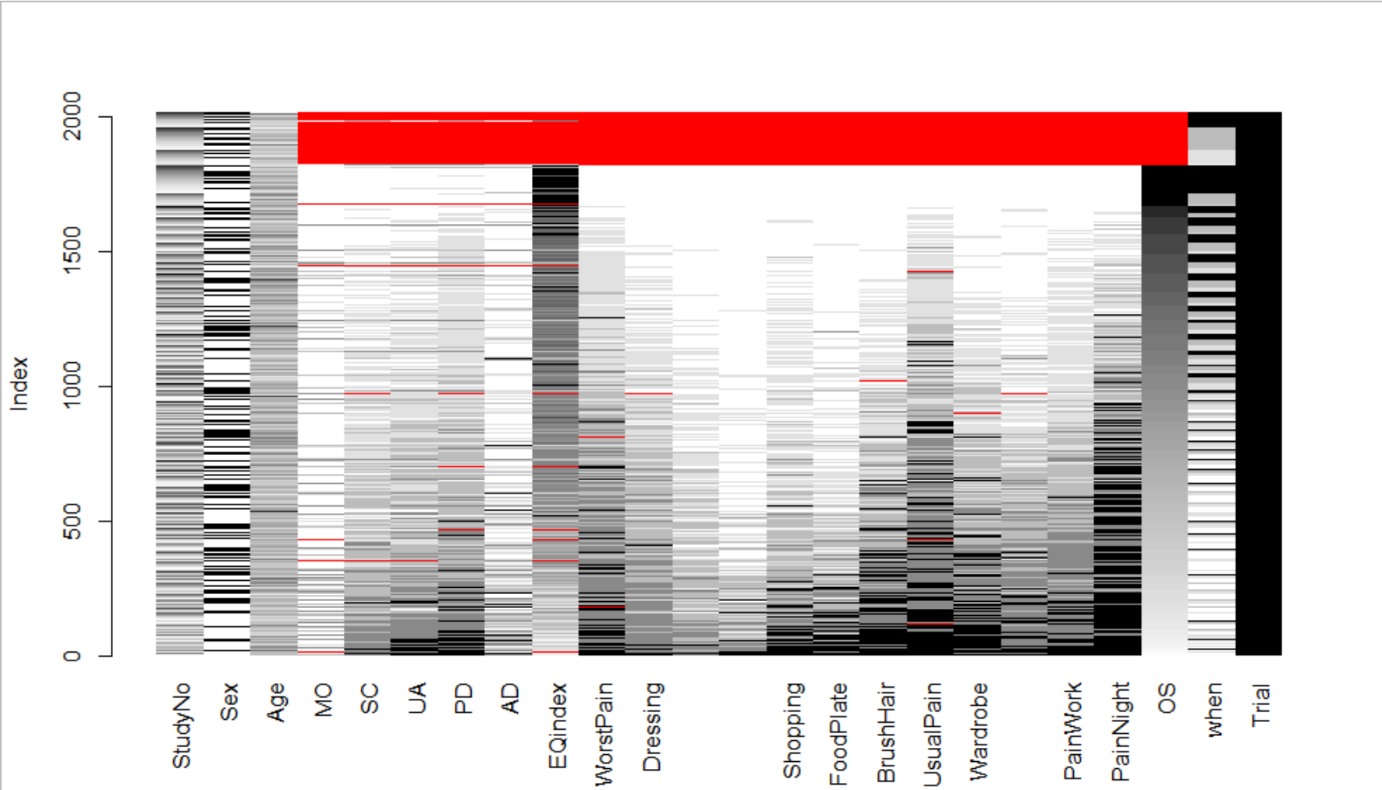


### Missingness relationships between EQ-5D and OSS

The below plot demonstrates similar distributions of EQ-5D index or OSS score when either is missing, as shown by the overlapping box plots. Red indicates missing, blue indicates present.


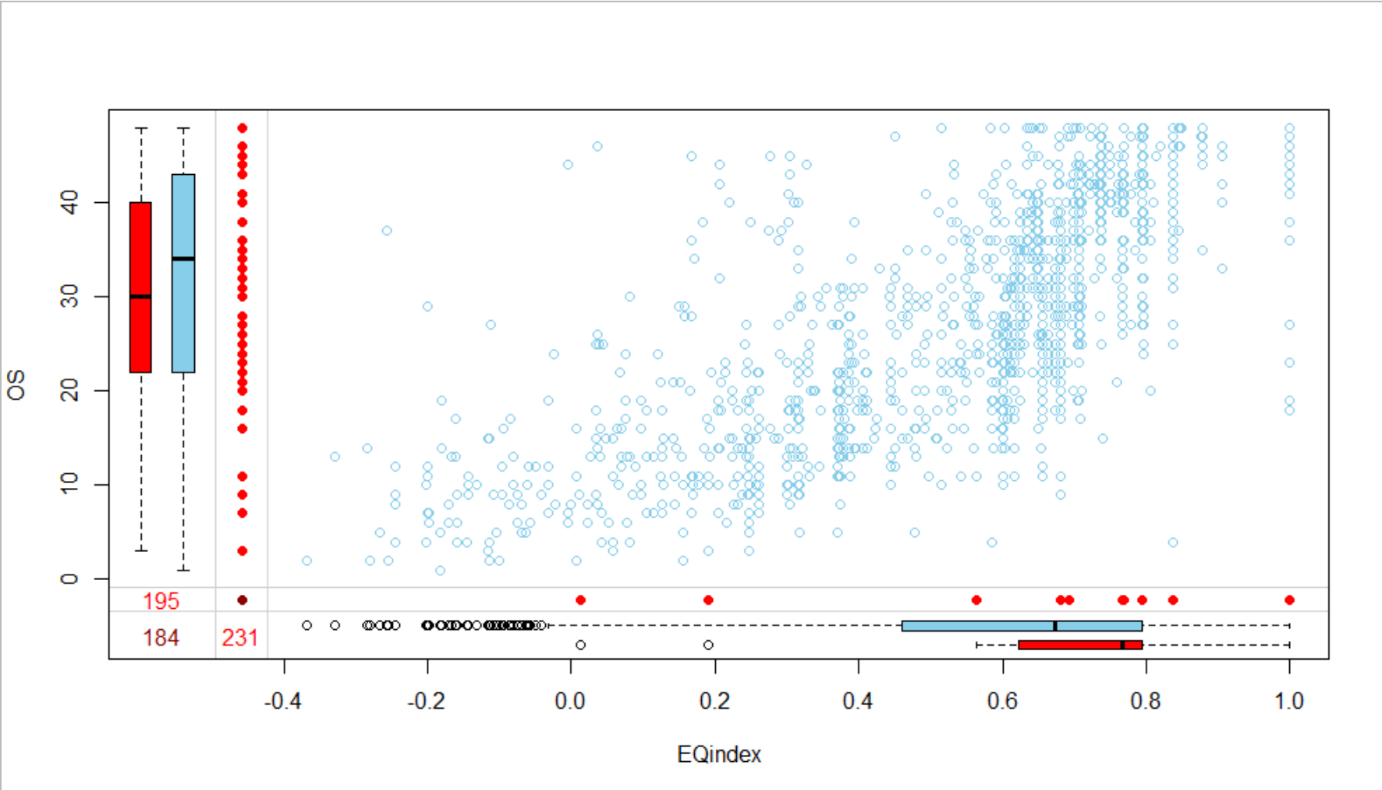


Distributions of EQ-5D index with and without missing OSS values. NA=missing; !NA=not missing.


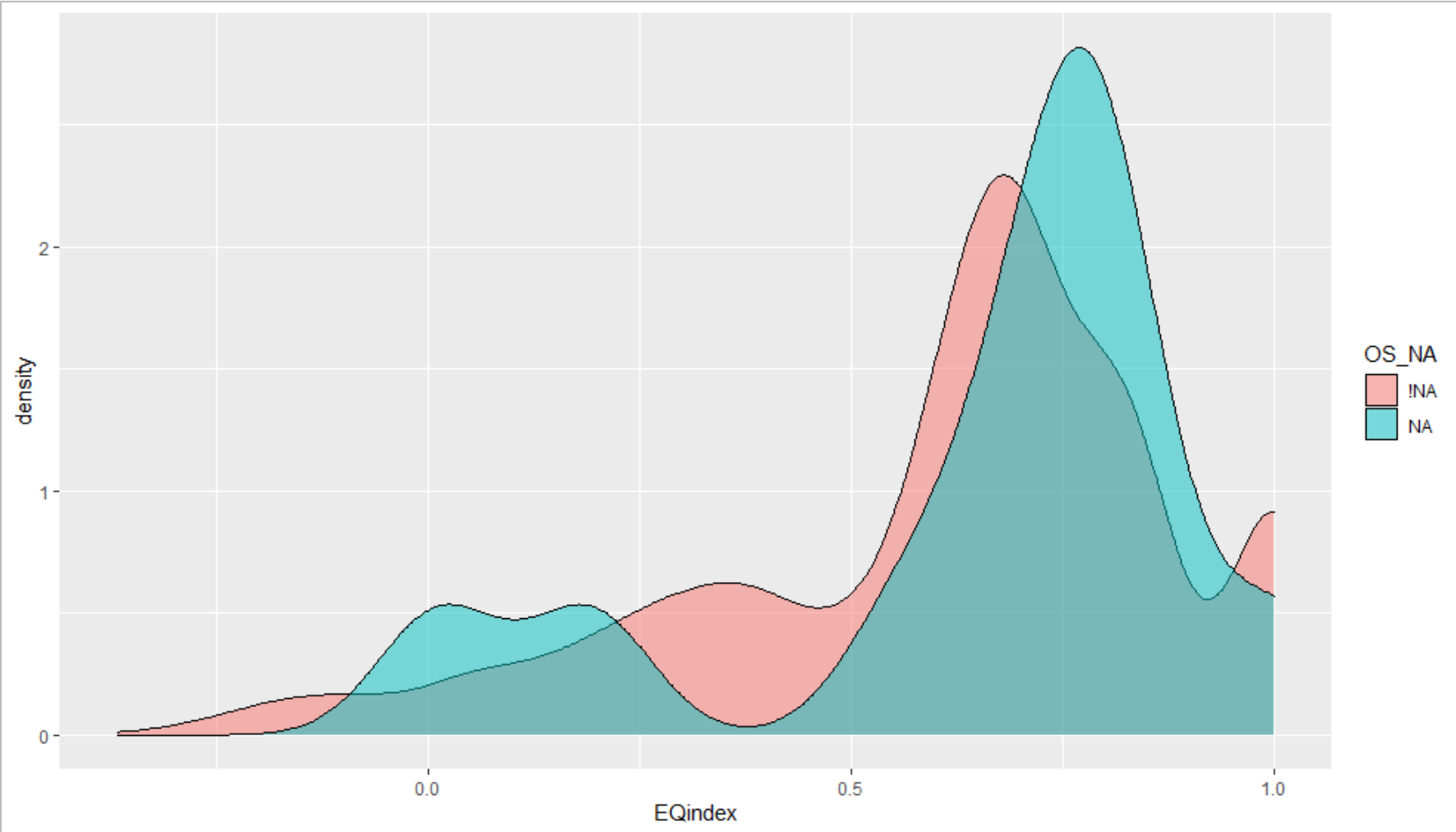


Distributions of OSS with and without missing EQ-5D index values. NA=missing; !NA=not missing.


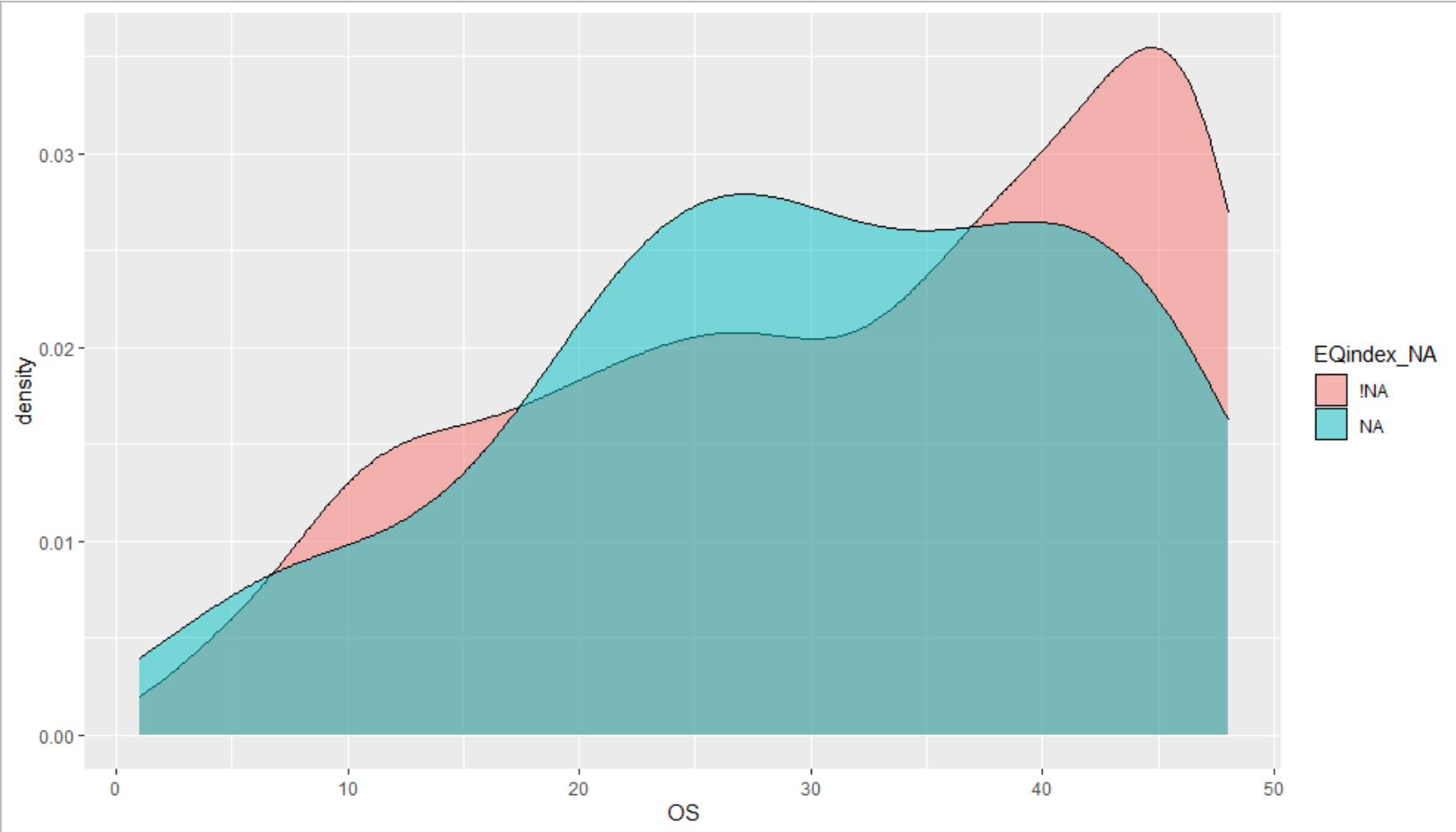


## Completely missing questionnaire responses

### EQ-5D index

The below histograms and box plots compare the baseline distributions of EQ-5D health index for patients who either had no missing questionnaire responses, or who had at least one completely missing questionnaire response over the study follow-up period. There does not appear to be a statistically significant difference in the baseline EQ-5D values between these two groups.


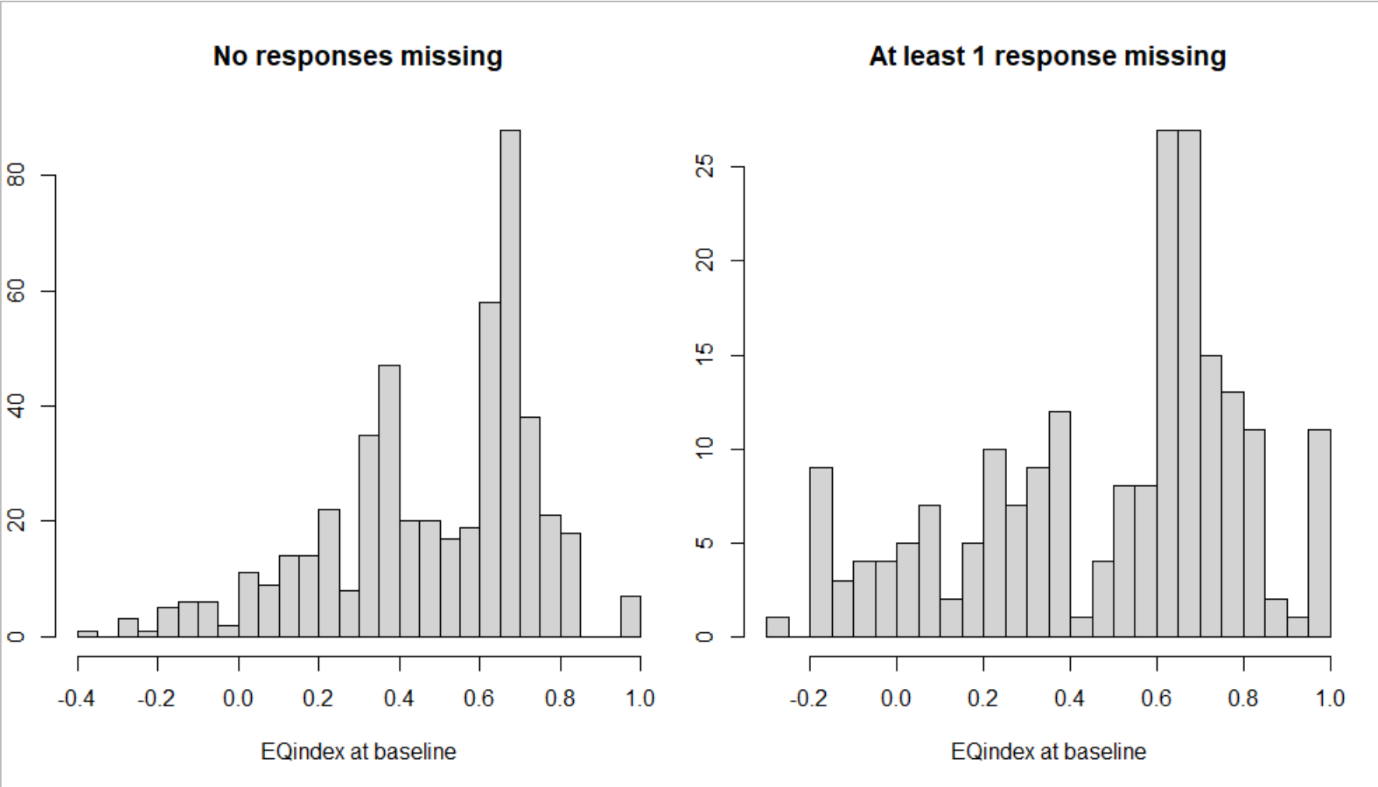


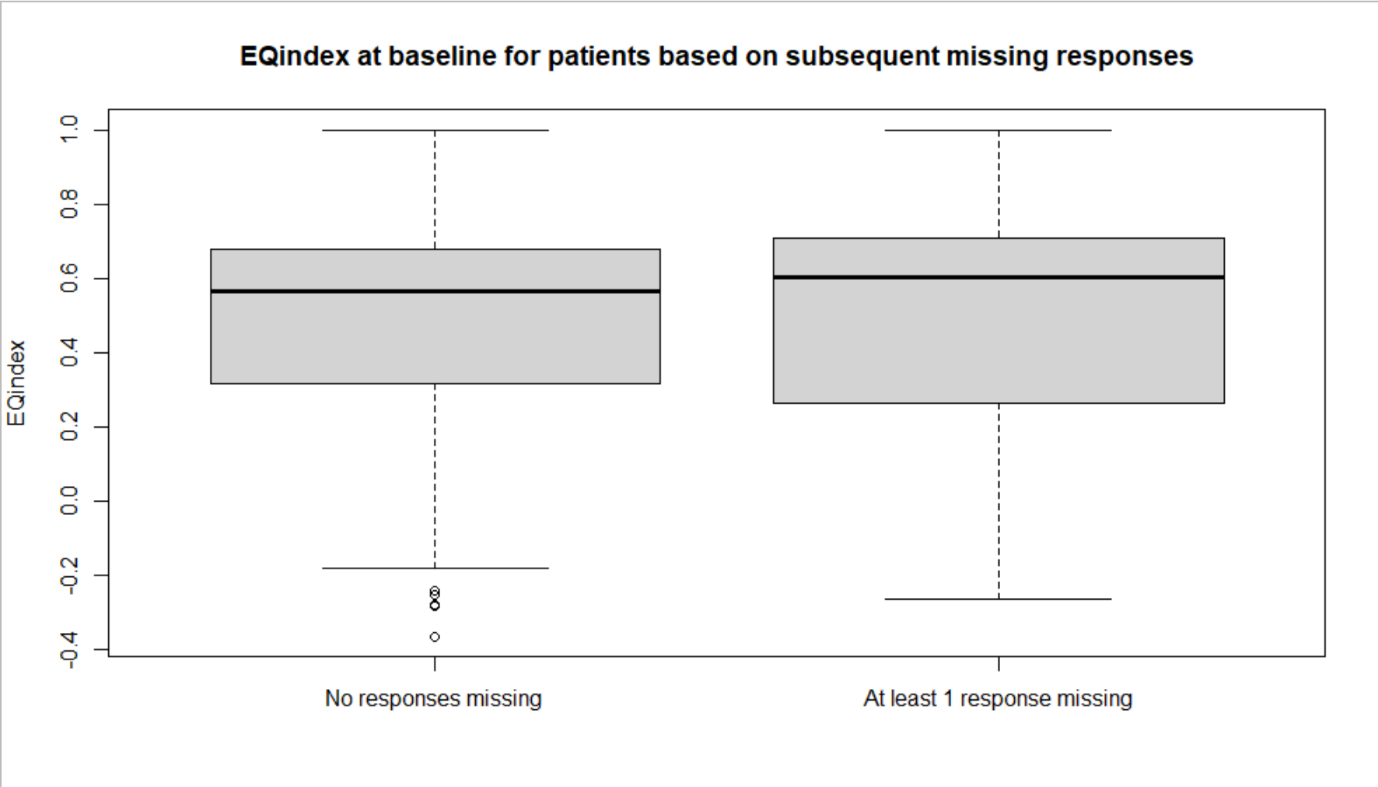


Mann-Whitney U-test: 0.352

### OSS score

The below histograms and box plots compare the baseline distributions of OSS scores for patients who either had no missing questionnaire responses, or who had at least one completely missing questionnaire response over the study follow-up period. There does not appear to be a statistically significant difference in the baseline OSS scores between these two groups.

(OS: Oxford Shoulder Score)


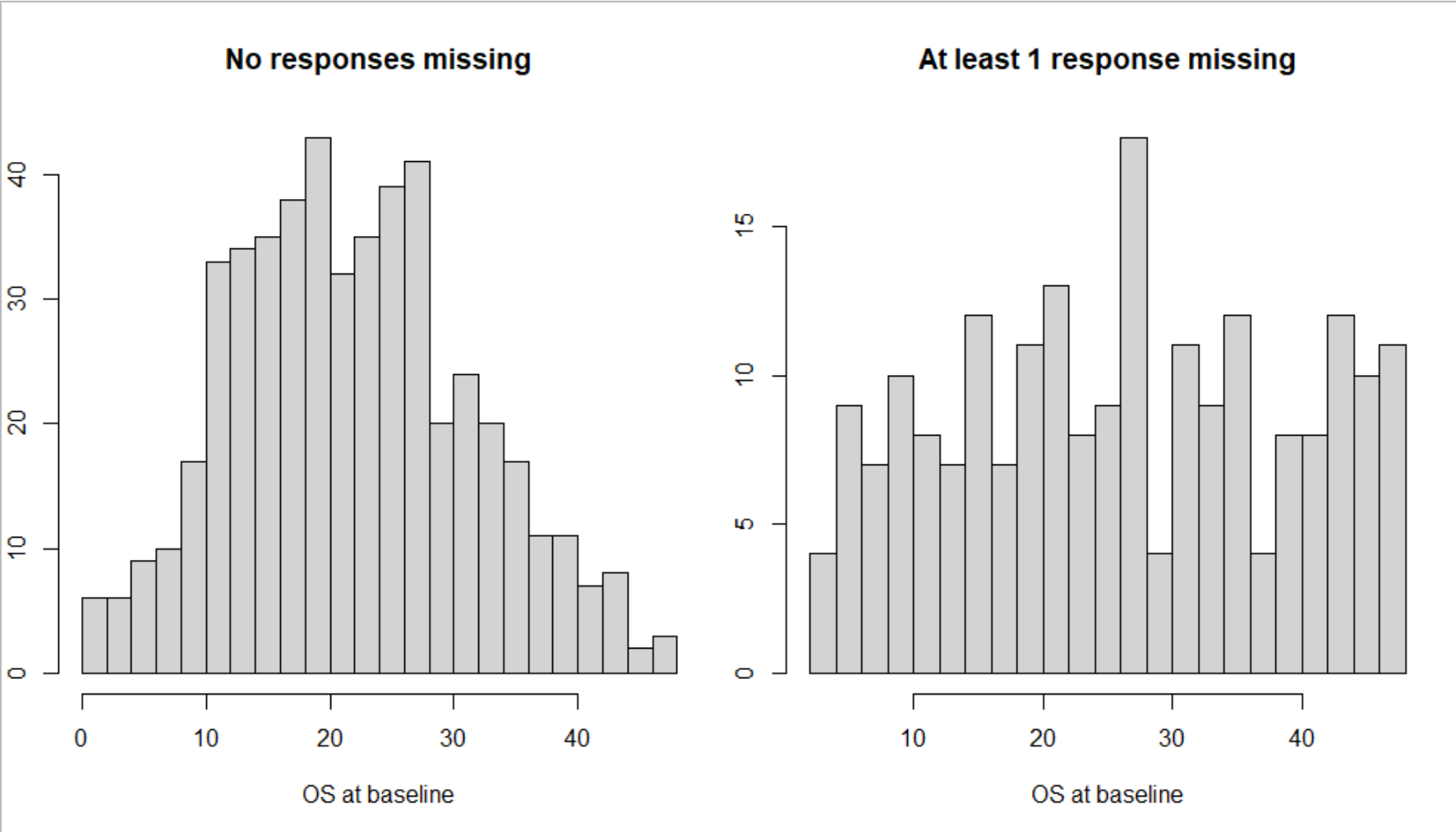


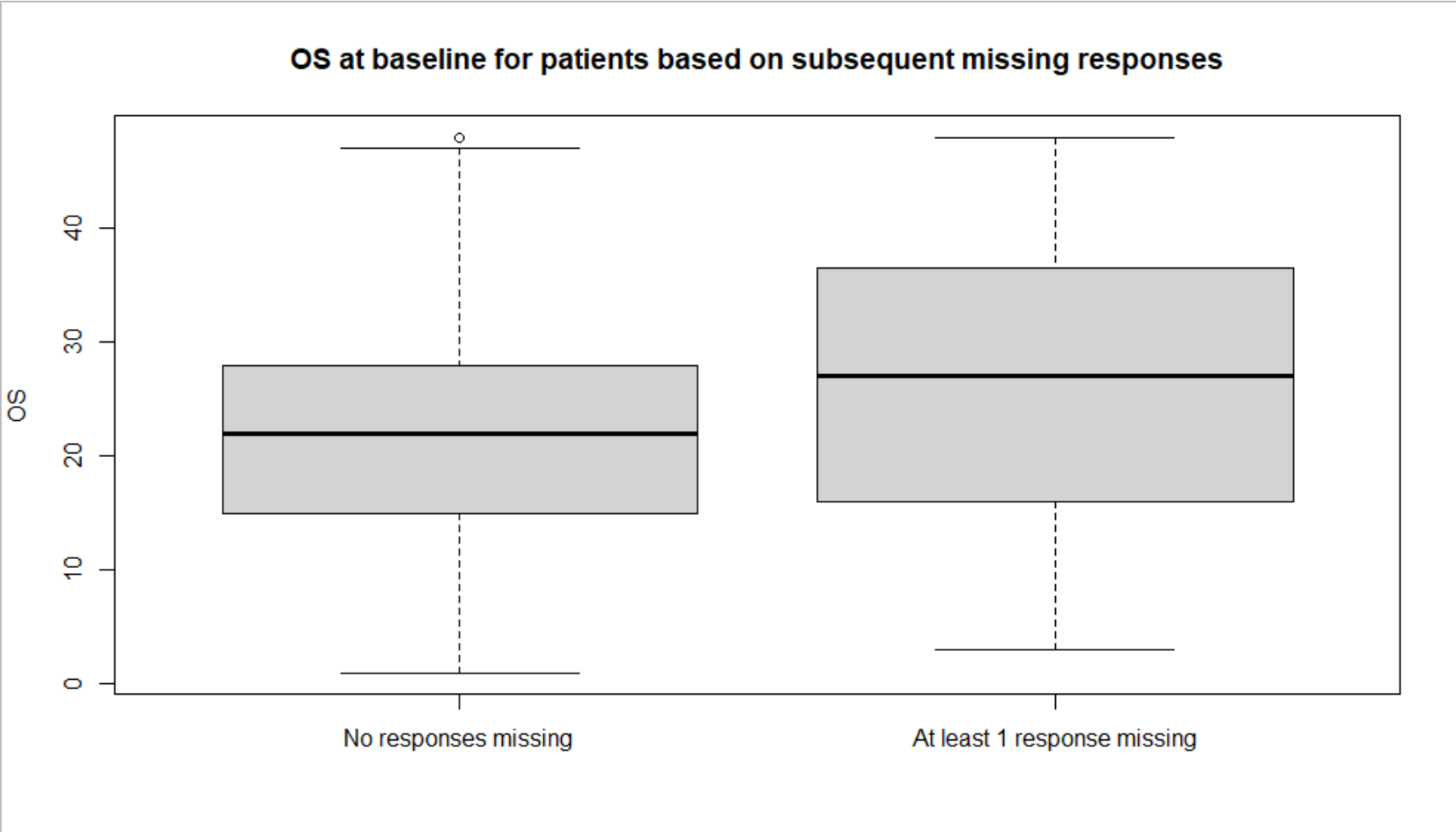


Mann-Whitney U-test: 0.123

# Section 3: Description of models

## TTU regression models

### Univariate linear

The simplest model (univariate linear model) was an ordinary least-squares regression using just the total OSS to predict the health index. We used the *lm* function in R to produce the univariate model that is described by Equation 1.


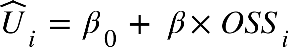
 Eq.1

where
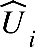
 is the predicted health index,
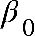
 is the constant term,
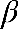
 is the vector of the regressor, and
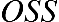
 is the total OSS score.

### Linear splines

We evaluated the inclusion of a piecewise function to the above model by introducing the possibility of the linear model to have knots. The number of knots investigated was between one and three for practical application and computational simplicity. Adjacent linear segments were allowed to vary in slope but were required to be continuous with one another to reflect the continuity in disease severity. The knot values were chosen as those that improved model fit by minimising the mean of the squares of the residuals. The function of a linear model consisting of a single knot was implemented by adding the *knots* parameter in the *lm* function and is described in Equation 2.


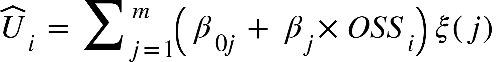
 Eq.2

where
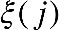
is an indicator function- it is zero except for the range of values of OSS where it is equal to 1. *m* is the number of knots plus one and *j* is the segment descriptor.

### Polynomial

We evaluated polynomial univariate models, where the relationship between OSS and the health index was modelled as an *n*^th^ degree polynomial in OSS, as described by Equation 4.


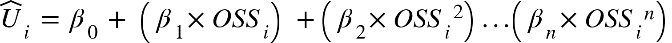
 Eq.4

where *n* is the *n*^th^ term of the model. We evaluated a squared and cubic polynomial model. The cubic polynomial model consisted of a constant, linear, square, and cubic term.

### Cubic splines

We evaluated the inclusion of a piecewise function to the cubic model by introducing the possibility of the model to have knots, as in the linear models above.

### Multivariable linear

The second model that was evaluated was a multivariable linear regression model where all available OSS question responses were used to predict the health index, described by equation 3.


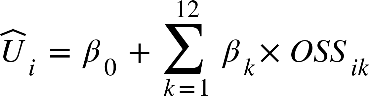
 Eq.3

where *k* is each of the 12 OSS question responses. We checked for the presence of collinearity between variables using the *olsrr* package in R to check the variance inflation factor statistic and condition indices ^2^. There was evidence of a moderate amount of collinearity (although the variance inflation factor for all variables was less than 5.5 and the condition index was less than 30).

### Two-part

The first component (logistic) was developed to predict which patients were expected to have a health index of 1, as shown in Equation 5.


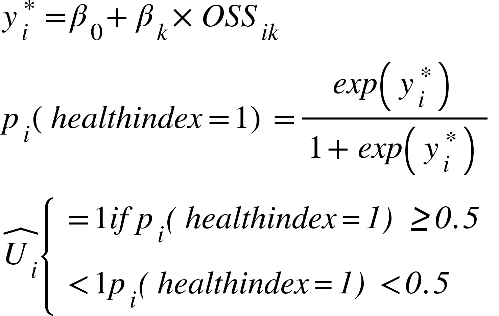
 Eq.5

where
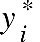
is an unobserved latent variable representing the log odds of the health index being equal to one. The second component predicted the health index of those patients not predicted to score 1 by the first component. These were subsequently combined so that if the logistic component’s predicted probability was greater or equal to 0.5, this was assigned a value of 1. If the predicted probability was less than 0.5, the linear component’s prediction was assigned instead.

### Tobit

Tobit regression models, also called censored regression models, are designed to estimate linear relationships between variables when there is left- or right-censoring in the dependent variable ^3^. We developed a tobit model using the total OSS score, where the predicted health index was censored at values of -0.594 and 1, corresponding to the UK EQ-5D-3L tariff ^4^.

### Adjusted Limited Dependent Variable Mixture Model (ALDVMM)

We used the *aldvmm* package in R and followed published guidance in developing this mixture model ^5,6^. Fitting a multivariate model that included all 12 OSS question responses while ensuring the mixture model converged and produced comparable predictions of the testing data proved impossible, so we only report a model that uses the total OSS as the single regressor.

## Response mapping models

### Ordered logistic model

Responses to EQ-5D questions are ordered, but the parallel regression assumption must hold in order to use an ordered logistic model. Using the *brant* command in R, we confirmed that the parallel regression assumption did hold for all five EQ-5D domains ^7^. Equation 6 describes the ordered logistic regression model


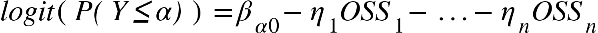
 Eq.6

where
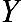
 is the ordinal EQ-5D question response with
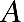
 categories. Then
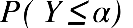
is the cumulative probability of
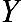
 less than or equal to a specific category
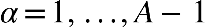
. At this step, Gray et al used a Monte Carlo procedure rather than taking the most likely predicted value ^8^. However, they found that these simulations produced very small differences given their sample size, so they only reported a single simulation. Given our sample size we decided to also assign response categories after only one simulation.

### Seemingly unrelated regression (SUR)

Response mapping for various models often consists of more than one equation. In this instance, there is an equation for each EQ-5D domain. The disturbance terms of these equations may be contemporaneously correlated which, if unaccounted for, can lead to inefficient estimates of the coefficients. SUR estimates all equations simultaneously with a generalised least squares estimator, leading to efficient estimates ^9^. We used the *systemfit* package in R to develop a SUR model where all five EQ-5D domain responses were predicted simultaneously ^10^.

# Section 4: Model specifications

All model parameters reported below were calculated using the entire dataset.

## Univariate linear

Table 4.1: OLS model with total OSS score. Number of observations= 1732. R2= 0.635. Adjusted R2=0.6347. F-statistic = 1536, p-value <0.001.

|  | Coefficient | Standard error | p value |
| --- | --- | --- | --- |
| Total OSS score | 0.01757 | 0.0004483 | <0.001 |
| Constant | 0.05901 | 0.0170440 | 0.01 |

Application of model:

Enter the OSS score of the patient in the following equation. The equation will output the predicted EQ-5D index:

Predicted EQ-5D index =0. 05901+ 0. 01757*OSS score

## Linear splines

Model summary

Knots:

imin= **21**

jmin= **42**

Coefficients:

Estimate Std. Error p value

(Intercept) **-0.08407** 0.01937 <0.001

bs(OSS, knots = c(imin, jmin), degree = 1)1 **0.59187** 0.02432 < 0.001

bs(OSS, knots = c(imin, jmin), degree = 1)2 **0.82586** 0.02004 < 0.001

bs(OSS, knots = c(imin, jmin), degree = 1)3 **1.01607** 0.02247 < 0.001

Linear splines model plot:


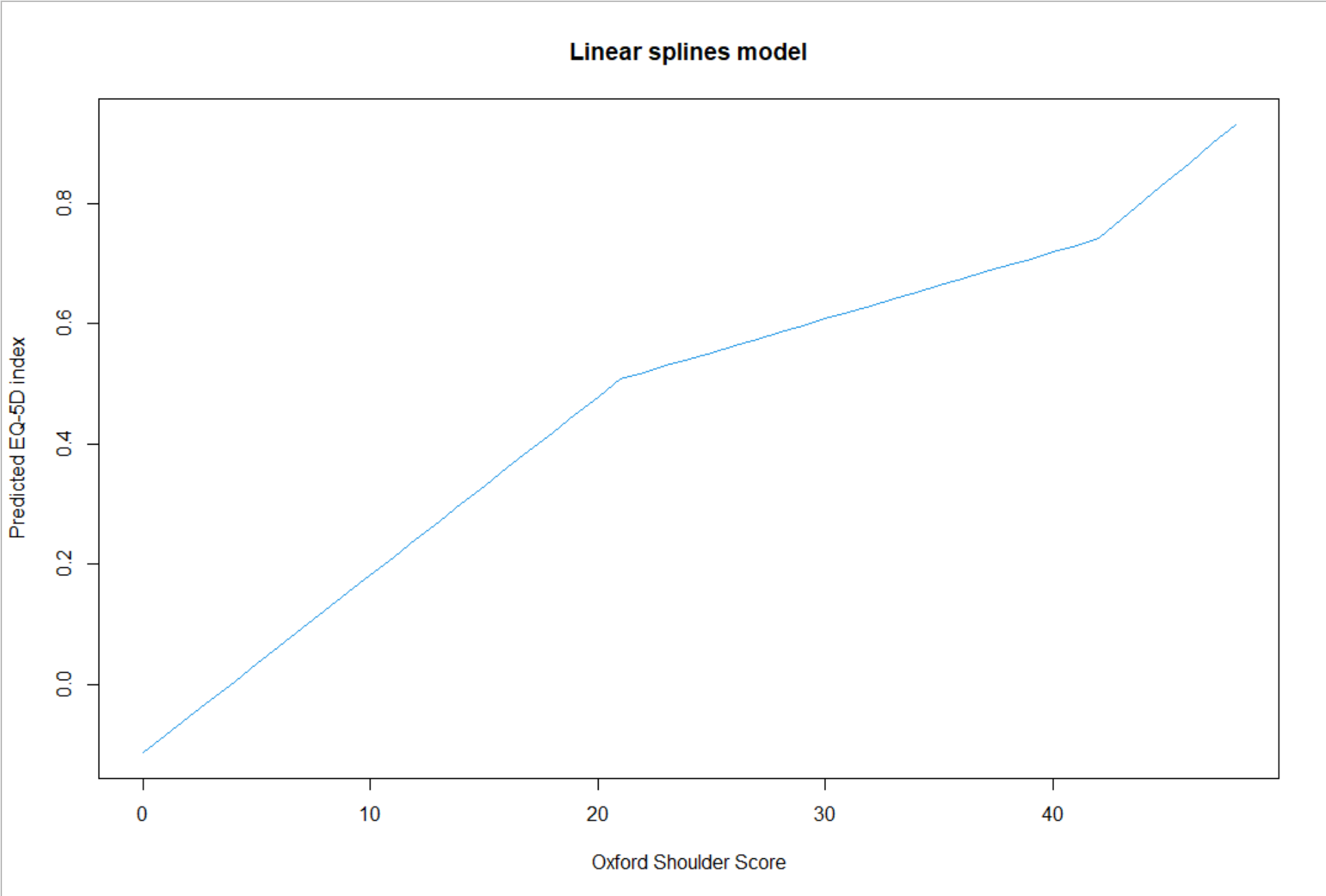


Application of model:

Enter the OSS score of the patient in one of the three following equations depending on the range within which the score lies. The equation will output the predicted EQ-5D index as corresponds to the above linear splines model graph.

Predicted EQ-5D index = -0.11367 + 0.0296*OSS score (valid for 0≤OSS≤21)

Predicted EQ-5D index = 0.2738 + 0.0111*OSS score (valid for 21<OSS≤42)

Predicted EQ-5D index = -0.5897 + 0.0317*OSS score (valid for 42<OSS≤48)

## Polynomial (cubic)

Table 4.2: Polynomial (cubic) model with total OSS. Number of observations= 1732. R2= 0.6412. Adjusted R2=0.6408. F-statistic = 860, p-value <0.001.

|  | Coefficient | Standard error | p value |
| --- | --- | --- | --- |
| Total OSS score (OSS) | 0.06245 | 0.007005 | <0.001 |
| OSS^2^ | -0.001669 | 0.0002577 | <0.001 |
| OSS^3^ | 0.00001838 | 0.000002895 | <0.001 |
| Constant | -0.2679 | 0.05704 | <0.001 |

Application of model:

Enter the OSS score of the patient in the following equation. The equation will output the predicted EQ-5D index:

Predicted EQ-5D index = -0. 2679+ 0. 06245*OSS score – 0. 001669*OSS score^2^ + 0. 00001838*OSS score^3^

## Polynomial (squared)

Details of the polynomial (squared) model are provided below. Following sequential likelihood ratio tests, the cubic model was found to be superior, so we do not report on the squared model further.

Table 4.3: Polynomial (squared) model with total OSS. Number of observations= 1732. R2= 0.6412. Adjusted R2=0.6408. F-statistic = 860, p-value <0.001.

|  | Coefficient | Standard error | p value |
| --- | --- | --- | --- |
| Total OSS score (OSS) | 0.0262447 | 0.0021609 | <0.001 |
| OSS^2^ | -0.0001507 | 0.0000344 | <0.001 |
| Constant | -0.0408175 | 0.03195 | 0.204 |

## Cubic splines

Knot:

imincub= **23**

Coefficients:

Estimate Std. Error p value

(Intercept) **-0.06477** 0.04220 0.125

bs(OSS, knots = c(imincub), degree = 3)1 **0.15773** 0.06552 0.016

bs(OSS, knots = c(imincub), degree = 3)2 **0.81058** 0.04064 <0.001

bs(OSS, knots = c(imincub), degree = 3)3 **0.66446** 0.05151 <0.001

bs(OSS, knots = c(imincub), degree = 3)4 **1.00000**  0.04249 <0.001

Cubic splines model plot:


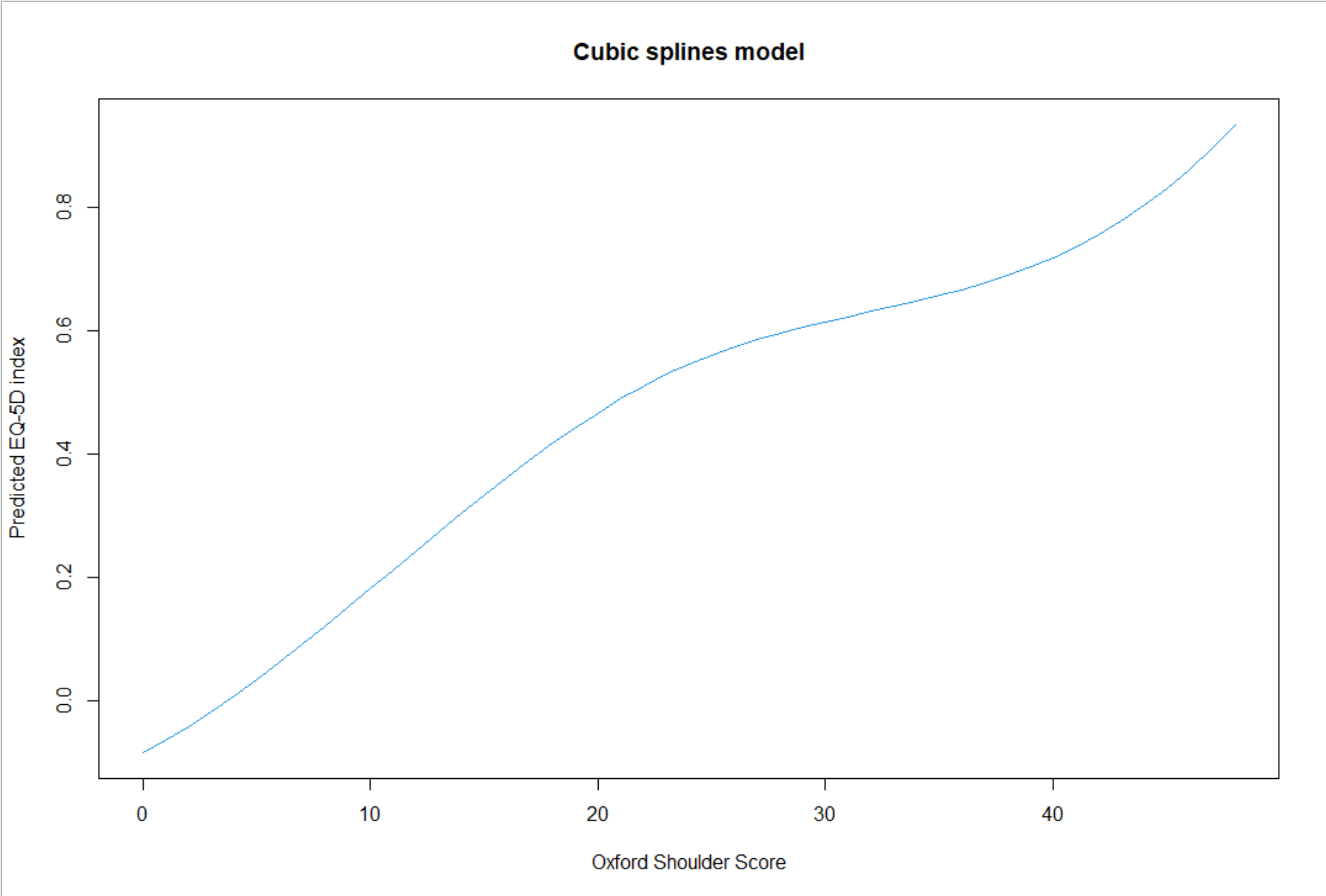


Application of model:

Enter the OSS score of the patient in one of the three following equations depending on the range within which the score lies. The equation will output the predicted EQ-5D index as corresponds to the above cubic splines model graph.

Predicted EQ-5D index = -0.08533 + 0.01959*OSS +0.001007*OSS^2^ -0.00003033*OSS^3^ (valid for 0≤OSS≤23)

Predicted EQ-5D index = -0.9521 + 0.1326*OSS - 0.003908*OSS^2^ + 0.0000409*OSS^3^ (valid for 23<OSS≤48)

## Multivariable linear

Table 4.4: Multivariable linear model. Number of observations= 1732. Multiple R-squared: 0.6539. Adjusted R-squared: 0.6521. F-statistic= 213, p-value: <0.001

|  | Coefficient | Standard Error | P value |
| --- | --- | --- | --- |
| Constant | 0.013157 | 0.028153 | 0.6411 |
| OSS1: How would you describe the worst pain you had from your shoulder? | 0.017953 | 0.007271 | 0.0143 |
| OSS2: Have you had any trouble dressing yourself because of your shoulder? | 0.031889 | 0.008907 | <0.001 |
| OSS3: Have you had any trouble getting in and out of a car or using public transport because of your shoulder? | Excluded |  |  |
| OSS4: Have you been able to use a knife and fork at the same time? | 0.028569 | 0.010988 | 0.0102 |
| OSS5: Could you do household shopping on your own? | 0.046794 | 0.008010 | <0.001 |
| OSS6: Could you carry a tray containing a plate of food across the room? | 0.028035 | 0.008060 | <0.001 |
| OSS7: Could you brush/comb your hair with the affected arm? | 0.003183 | 0.006624 | 0.6313 |
| OSS8: How would you describe the pain you usually had from your shoulder? | 0.017892 | 0.005440 | 0.001 |
| OSS9: Could you hang your clothes up in the wardrobe using the affected arm? | Excluded |  |  |
| OSS10: Have you been able to wash and dry yourself under both arms? | Excluded |  |  |
| OSS11: How much has the pain from your shoulder interfered with your usual work (including housework)? | 0.037333 | 0.008802 | <0.001 |
| OSS12: Have you been troubled by pain from your shoulder in bed at night? | 0.009739 | 0.004833 | 0.0451 |

Application of model:

Enter the patient’s response value (0,1,2,3 or 4) for each OSS question in the following equation. The equation will output the predicted EQ-5D index:

Predicted EQ-5D index =0. 013157+ 0. 017953*OSS1 + 0. 031889*OSS2 + 0. 028569*OSS4 + 0. 046794*OSS5 + 0. 028035*OSS6 + 003183*OSS7 + 0. 017892*OSS8 + 0. 037333*OSS11 + 0. 009739*OSS12

## Two-part

**Logistic component (part one):**

Table 4.5a: Coefficients for logistic component of the two-part model. AIC: 660.26

|  | Coefficient | Standard error | P value |
| --- | --- | --- | --- |
| (Intercept) | -21.46 | 1.68087 | <0.001 |
| OS | 0.4578 | 0.03689 | <0.001 |

**Linear component (part two):**

Table 4.5b: Coefficients for linear component of the two-part model. Multiple R-squared: 0.5662, Adjusted R-squared: 0.5659, F-statistic: 2001, p-value: < 0.001

|  | Coefficient | Standard error | P value |
| --- | --- | --- | --- |
| (Intercept) | 0.0905 | 0.0114094 | <0.001 |
| OS | 0.0159 | 0.0003566 | <0.001 |

## Tobit

Table 4.6: Tobit model using total OSS score. Log-likelihood = 248. a: The log standard deviation of the latent variable. b: the expectation of the latent uncensored normal variables (the usual intercept of the model).

|  | Coefficient | Standard error | p value |
| --- | --- | --- | --- |
| Total OSS score | 0.0188536 | 0.0003553 | <0.001 |
| Constant (log(σ))^a^ | -1.6957046 | 0.0182072 | <0.001 |
| Constant (μ)^b^ | 0.0319397 | 0.0119428 | 0.007 |

## ALDVMM

Table 4.7: Coefficients for ALDVMM model. Application of this model requires that it is run with a statistical package such as aldvmm in R.

|  |  | Estimate | Std. Err. | z | P>\|z\| | [95% Conf. | Interval] |
| --- | --- | --- | --- | --- | --- | --- | --- |
| E[y\|X, c] |  |  |  |  |  |  |  |
| Comp1 | (Intercept) | 0.354 | 0.030 | 11.912 | 0 | 0.296 | 0.412 |
|  | OS | 0.001 | 0.002 | 0.542 | 0.588 | -0.003 | 0.006 |
|  | lnsigma | -1.128 | 0.058 | -19.360 | 0 | -1.242 | -1.014 |
| Comp2 | (Intercept) | 0.119 | 0.011 | 10.629 | 0 | 0.097 | 0.140 |
|  | OS | 0.016 | 0 | 48.922 | 0 | 0.016 | 0.017 |
|  | lnsigma | -2.063 | 0.027 | -75.342 | 0 | -2.117 | -2.009 |
|  |  |  |  |  |  |  |  |
| P[c\|X] |  |  |  |  |  |  |  |
| Comp1 | (Intercept) | 0.533 | 0.216 | 2.473 | 0.013 | 0.111 | 0.956 |
|  | OS | -0.110 | 0.015 | -7.275 | 0 | -0.139 | -0.080 |
|  |  |  |  |  |  |  |  |
|  |  |  |  |  |  |  |  |
| N = 1732 | ll = 324.42 | AIC = -632.84 | BIC = -592.09 | |  |  |  |

## Ordered logistic regression

Table 4.8: Ordered logistic regression model coefficients. 1|2, 2|3, 3|4 and 4|5 are the values of the constants for each logit estimated model giving the cumulative probability that the response is less than or equal to 1, 2, 3, and 4 respectively.

|  | Mobility | | | Self-Care | | | Usual Activities | | | Pain and Discomfort | | | Anxiety and Depression | | |
| --- | --- | --- | --- | --- | --- | --- | --- | --- | --- | --- | --- | --- | --- | --- | --- |
|  | Coefficient | Standard error | p value | Coefficient | Standard error | p value | Coefficient | Standard error | p value | Coefficient | Standard error | p value | Coefficient | Standard error | p value |
| OSS1: How would you describe the worst pain you had from your shoulder? | 0.414615 | 0.106346 | 3.898753 | 0.083037 | 0.08456 | 0.981991 | -0.04618 | 0.081049 | -0.56976 | -0.67995 | 0.084517 | -8.04506 | -0.00668 | 0.083744 | -0.07979 |
| OSS2: Have you had any trouble dressing yourself because of your shoulder? | -0.16231 | 0.115616 | -1.40385 | -1.06912 | 0.100284 | -10.661 | -0.28591 | 0.093091 | -3.07124 | -0.09772 | 0.095864 | -1.01936 | 0.028869 | 0.094117 | 0.306735 |
| OSS3: Have you had any trouble getting in and out of a car or using public transport because of your shoulder? | -0.15538 | 0.101596 | -1.52938 | -0.16959 | 0.087276 | -1.94321 | -0.11521 | 0.085979 | -1.34002 | -0.06218 | 0.086127 | -0.72195 | -0.12441 | 0.08326 | -1.49428 |
| OSS4: Have you been able to use a knife and fork – at the same time? | -0.0257 | 0.091286 | -0.28158 | -0.17791 | 0.085732 | -2.07519 | -0.08631 | 0.084189 | -1.0252 | -0.13624 | 0.085907 | -1.58591 | -0.17978 | 0.079907 | -2.24991 |
| OSS5: Could you do household shopping on your own? | -0.4573 | 0.089139 | -5.13016 | -0.46986 | 0.076685 | -6.12717 | -0.64505 | 0.076517 | -8.43014 | -0.35662 | 0.077558 | -4.59814 | -0.42515 | 0.074301 | -5.72202 |
| OSS6: Could you carry a tray containing a plate of food across the room? | -0.38751 | 0.084827 | -4.56829 | -0.07255 | 0.077266 | -0.93892 | -0.09447 | 0.076661 | -1.23235 | -0.2072 | 0.077012 | -2.69052 | -0.14174 | 0.073199 | -1.93635 |
| OSS7: Could you brush/comb your hair with the affected arm? | 0.014079 | 0.09796 | 0.143727 | -0.18301 | 0.077418 | -2.36391 | -0.16548 | 0.076476 | -2.16375 | -0.04719 | 0.079497 | -0.5936 | 0.055415 | 0.07819 | 0.708718 |
| OSS8: How would you describe the pain you usually had from your shoulder? | -0.04705 | 0.091309 | -0.51525 | -0.0976 | 0.072739 | -1.34183 | -0.08286 | 0.071228 | -1.16326 | -0.65501 | 0.074718 | -8.76638 | 0.046308 | 0.074718 | 0.619771 |
| OSS9: Could you hang your clothes up in the wardrobe using the affected arm? | 0.194911 | 0.099069 | 1.967415 | 0.079324 | 0.080003 | 0.991503 | 0.019075 | 0.078503 | 0.242988 | 0.131403 | 0.081077 | 1.620712 | 0.092844 | 0.080426 | 1.154396 |
| OSS10: Have you been able to wash and dry yourself under both arms? | 0.177234 | 0.095866 | 1.848764 | -0.42905 | 0.08089 | -5.30405 | 0.035176 | 0.079554 | 0.44217 | 0.033082 | 0.081372 | 0.406555 | -0.05282 | 0.080554 | -0.65569 |
| OSS11: How much has the pain from your shoulder interfered with your usual work (including housework)? | -0.24316 | 0.115998 | -2.09628 | -0.10564 | 0.094317 | -1.12004 | -1.02339 | 0.095279 | -10.741 | -0.38443 | 0.094854 | -4.05283 | -0.42395 | 0.093405 | -4.5388 |
| OSS12: Have you been troubled by pain from your shoulder in bed at night? | -0.00217 | 0.078644 | -0.02765 | -0.09289 | 0.063079 | -1.47254 | -0.11734 | 0.060014 | -1.95525 | -0.49669 | 0.063796 | -7.7856 | 0.039995 | 0.062804 | 0.636819 |
| 1\|2 | -1.00617 | 0.240546 | -4.18287 | -9.0826 | 0.344468 | -26.3671 | -8.99892 | 0.330916 | -27.1939 | -10.8537 | 0.363643 | -29.8472 | -2.92365 | 0.225628 | -12.9578 |
| 2\|3 | -0.34055 | 0.239305 | -1.42309 | -6.13686 | 0.297119 | -20.6546 | -6.15178 | 0.292544 | -21.0286 | -6.93695 | 0.305373 | -22.7163 | -1.65225 | 0.216212 | -7.6418 |
| 3\|4 | 0.882584 | 0.24704 | 3.572632 | -3.01043 | 0.242589 | -12.4096 | -3.11898 | 0.240123 | -12.9891 | -3.64729 | 0.250914 | -14.536 | -0.02104 | 0.215744 | -0.09751 |
| 4\|5 | 4.124772 | 0.54556 | 7.560614 | 0.415273 | 0.303475 | 1.368393 | -0.38084 | 0.234482 | -1.62417 | -0.74038 | 0.226864 | -3.26353 | 1.07948 | 0.242093 | 4.458939 |

## SUR

Table 4.9a: Model for EQ-5D (Mobility)

|  | Coefficient | Standard error | p value |
| --- | --- | --- | --- |
| Constant | 2.402997 | 0.088195 | 27.2463 |
| OSS1: How would you describe the worst pain you had from your shoulder? | 0.126617 | 0.031967 | 3.960844 |
| OSS2: Have you had any trouble dressing yourself because of your shoulder? | -0.07333 | 0.037394 | -1.96091 |
| OSS3: Have you had any trouble getting in and out of a car or using public transport because of your shoulder? | -0.02697 | 0.034044 | -0.79218 |
| OSS4: Have you been able to use a knife and fork – at the same time? | -0.04631 | 0.033534 | -1.38087 |
| OSS5: Could you do household shopping on your own? | -0.16974 | 0.030139 | -5.63209 |
| OSS6: Could you carry a tray containing a plate of food across the room? | -0.13643 | 0.030478 | -4.47638 |
| OSS7: Could you brush/comb your hair with the affected arm? | -0.00089 | 0.030937 | -0.02881 |
| OSS8: How would you describe the pain you usually had from your shoulder? | -0.00776 | 0.027525 | -0.28189 |
| OSS9: Could you hang your clothes up in the wardrobe using the affected arm? | 0.074188 | 0.031367 | 2.365167 |
| OSS10: Have you been able to wash and dry yourself under both arms? | 0.051184 | 0.031626 | 1.618451 |
| OSS11: How much has the pain from your shoulder interfered with your usual work (including housework)? | -0.08479 | 0.036409 | -2.32889 |
| OSS12: Have you been troubled by pain from your shoulder in bed at night? | 0.013208 | 0.023588 | 0.559926 |

Table 4.9b: Model for EQ-5D (Self-Care)

|  | Coefficient | Standard error | p value |
| --- | --- | --- | --- |
| Constant | 4.528246 | 0.07088 | 63.88587 |
| OSS1: How would you describe the worst pain you had from your shoulder? | -0.00935 | 0.025691 | -0.36407 |
| OSS2: Have you had any trouble dressing yourself because of your shoulder? | -0.08726 | 0.030052 | -2.90354 |
| OSS3: Have you had any trouble getting in and out of a car or using public transport because of your shoulder? | -0.04771 | 0.027361 | -1.74387 |
| OSS4: Have you been able to use a knife and fork – at the same time? | -0.03713 | 0.02695 | -1.37776 |
| OSS5: Could you do household shopping on your own? | -0.22298 | 0.024222 | -9.20584 |
| OSS6: Could you carry a tray containing a plate of food across the room? | -0.03486 | 0.024494 | -1.4231 |
| OSS7: Could you brush/comb your hair with the affected arm? | -0.05183 | 0.024863 | -2.08475 |
| OSS8: How would you describe the pain you usually had from your shoulder? | -0.00927 | 0.022121 | -0.41885 |
| OSS9: Could you hang your clothes up in the wardrobe using the affected arm? | -0.00529 | 0.025209 | -0.2098 |
| OSS10: Have you been able to wash and dry yourself under both arms? | 0.011653 | 0.025417 | 0.458492 |
| OSS11: How much has the pain from your shoulder interfered with your usual work (including housework)? | -0.30346 | 0.029261 | -10.371 |
| OSS12: Have you been troubled by pain from your shoulder in bed at night? | -0.0362 | 0.018957 | -1.9093 |

Table 4.9c: Model for EQ-5D (Usual Activities)

|  | Coefficient | Standard error | p value |
| --- | --- | --- | --- |
| Constant | 4.378676 | 0.065146 | 67.21342 |
| OSS1: How would you describe the worst pain you had from your shoulder? | 0.025945 | 0.023613 | 1.098784 |
| OSS2: Have you had any trouble dressing yourself because of your shoulder? | -0.29663 | 0.027621 | -10.7392 |
| OSS3: Have you had any trouble getting in and out of a car or using public transport because of your shoulder? | -0.07101 | 0.025147 | -2.82375 |
| OSS4: Have you been able to use a knife and fork – at the same time? | -0.06616 | 0.02477 | -2.67107 |
| OSS5: Could you do household shopping on your own? | -0.14538 | 0.022262 | -6.53052 |
| OSS6: Could you carry a tray containing a plate of food across the room? | -0.02865 | 0.022513 | -1.27278 |
| OSS7: Could you brush/comb your hair with the affected arm? | -0.05539 | 0.022851 | -2.42383 |
| OSS8: How would you describe the pain you usually had from your shoulder? | -0.02169 | 0.020332 | -1.06675 |
| OSS9: Could you hang your clothes up in the wardrobe using the affected arm? | 0.027883 | 0.023169 | 1.203442 |
| OSS10: Have you been able to wash and dry yourself under both arms? | -0.12849 | 0.02336 | -5.50021 |
| OSS11: How much has the pain from your shoulder interfered with your usual work (including housework)? | -0.03527 | 0.026893 | -1.31135 |
| OSS12: Have you been troubled by pain from your shoulder in bed at night? | -0.01689 | 0.017424 | -0.96927 |

Table 4.9d: Model for EQ-5D (Pain/Discomfort)

|  | Coefficient | Standard error | p value |
| --- | --- | --- | --- |
| Constant | 4.620503 | 0.065971 | 70.03831 |
| OSS1: How would you describe the worst pain you had from your shoulder? | -0.17544 | 0.023912 | -7.337 |
| OSS2: Have you had any trouble dressing yourself because of your shoulder? | -0.0221 | 0.027971 | -0.79023 |
| OSS3: Have you had any trouble getting in and out of a car or using public transport because of your shoulder? | -0.03392 | 0.025466 | -1.33218 |
| OSS4: Have you been able to use a knife and fork – at the same time? | -0.04838 | 0.025084 | -1.92878 |
| OSS5: Could you do household shopping on your own? | -0.11557 | 0.022544 | -5.1263 |
| OSS6: Could you carry a tray containing a plate of food across the room? | -0.06719 | 0.022798 | -2.94708 |
| OSS7: Could you brush/comb your hair with the affected arm? | -0.02186 | 0.023141 | -0.94449 |
| OSS8: How would you describe the pain you usually had from your shoulder? | -0.16376 | 0.020589 | -7.95383 |
| OSS9: Could you hang your clothes up in the wardrobe using the affected arm? | 0.041973 | 0.023463 | 1.788904 |
| OSS10: Have you been able to wash and dry yourself under both arms? | -0.00209 | 0.023656 | -0.08816 |
| OSS11: How much has the pain from your shoulder interfered with your usual work (including housework)? | -0.09561 | 0.027234 | -3.51065 |
| OSS12: Have you been troubled by pain from your shoulder in bed at night? | -0.13298 | 0.017644 | -7.53699 |

Table 4.9e: Model for EQ-5D (Anxiety/Depression)

|  | Coefficient | Standard error | p value |
| --- | --- | --- | --- |
| Constant | 3.371138 | 0.095417 | 35.33062 |
| OSS1: How would you describe the worst pain you had from your shoulder? | 0.028396 | 0.034585 | 0.821043 |
| OSS2: Have you had any trouble dressing yourself because of your shoulder? | -0.00169 | 0.040455 | -0.04179 |
| OSS3: Have you had any trouble getting in and out of a car or using public transport because of your shoulder? | -0.06211 | 0.036832 | -1.68635 |
| OSS4: Have you been able to use a knife and fork – at the same time? | -0.136 | 0.03628 | -3.74871 |
| OSS5: Could you do household shopping on your own? | -0.19616 | 0.032607 | -6.01586 |
| OSS6: Could you carry a tray containing a plate of food across the room? | -0.08444 | 0.032973 | -2.56098 |
| OSS7: Could you brush/comb your hair with the affected arm? | 0.000721 | 0.03347 | 0.021528 |
| OSS8: How would you describe the pain you usually had from your shoulder? | 0.020094 | 0.029779 | 0.674769 |
| OSS9: Could you hang your clothes up in the wardrobe using the affected arm? | 0.07857 | 0.033935 | 2.315307 |
| OSS10: Have you been able to wash and dry yourself under both arms? | -0.02811 | 0.034215 | -0.8217 |
| OSS11: How much has the pain from your shoulder interfered with your usual work (including housework)? | -0.16272 | 0.03939 | -4.13097 |
| OSS12: Have you been troubled by pain from your shoulder in bed at night? | 0.016061 | 0.02552 | 0.629364 |

# Section 5: Model performance

## Internal validation

Table 5.1: Summary model performance indicators- training sample (internal) validation. Performance is reported for the first training-testing (70:30) sample split, on the training sample.

|  | Mean fitted health index | Difference of means (observed-predicted) | Mean square error (MSE) | | | Mean absolute error (MAE) | | |
| --- | --- | --- | --- | --- | --- | --- | --- | --- |
|  |  |  | Total | OSS<median | OSS≥median | Total | OSS<median | OSS≥median |
| Univariate linear | 0.616689 | -4.44E-16 | 0.028421 | 0.037986 | 0.019471 | 0.124335 | 0.155106 | 0.095541 |
| Linear splines | 0.616689 | -3.33E-16 | 0.026753 | 0.037986 | 0.019471 | 0.11838 | 0.155106 | 0.095541 |
| Polynomial (cubic) | 0.616689 | 3.55E-15 | 0.027066 | 0.037986 | 0.019471 | 0.119857 | 0.155106 | 0.095541 |
| Cubic splines | 0.616689 | -1.11E-16 | 0.026755 | 0.037986 | 0.019471 | 0.118119 | 0.155106 | 0.095541 |
| Multivariable linear | 0.616689 | 4.44E-16 | 0.026978 | 0.037986 | 0.019471 | 0.121645 | 0.155106 | 0.095541 |
| Two-part | 0.613381 | 0.003308 | 0.029101 | 0.039123 | 0.019722 | 0.121527 | 0.159084 | 0.086381 |
| Tobit | 0.632178 | -0.01549 | 0.029 | 0.037472 | 0.021072 | 0.124748 | 0.15315 | 0.098169 |
| ALDVMM | 0.621632 | -0.00494 | 0.028548 | 0.037947 | 0.019752 | 0.124295 | 0.153511 | 0.096954 |
| Ordered logistic | 0.685403 | -0.06871 | 0.032852 | 0.043405 | 0.022976 | 0.118178 | 0.149942 | 0.088454 |
| SUR | 0.643648 | -0.02696 | 0.027816 | 0.037451 | 0.018799 | 0.109868 | 0.141214 | 0.080535 |

## 100-fold repeated random splitting of the training and testing samples

Table 5.2: Mean and standard deviation for each performance measure following repeated set.seed (1:100) random splits into training and testing datasets. Results are based on model performance on the testing samples.

| Model | Mean fitted health index | | Difference of means (observed-predicted) | | Mean square error (MSE) | | | | | | Mean absolute error (MAE) | | | | | |
| --- | --- | --- | --- | --- | --- | --- | --- | --- | --- | --- | --- | --- | --- | --- | --- | --- |
|  |  |  |  |  | Total | | OSS<median | | OSS≥median | | Total | | OSS<median | | OSS≥median | |
|  | mean | sd | mean | sd | mean | sd | mean | sd | mean | sd | mean | sd | mean | sd | mean | sd |
| Univariate linear | 0.613386 | 0.011139 | -0.00236 | 0.013125 | 0.028683 | 0.003021 | 0.039259 | 0.004078 | 0.018557 | 0.003913 | 0.123676 | 0.005342 | 0.156447 | 0.008027 | 0.092309 | 0.006708 |
| Linear splines | 0.613482 | 0.011557 | -0.00246 | 0.012418 | 0.027062 | 0.003096 | 0.039259 | 0.004078 | 0.018557 | 0.003913 | 0.11702 | 0.005176 | 0.156447 | 0.008027 | 0.092309 | 0.006708 |
| Polynomial (cubic) | 0.613429 | 0.011659 | -0.00241 | 0.01265 | 0.027304 | 0.003178 | 0.039259 | 0.004078 | 0.018557 | 0.003913 | 0.118332 | 0.005192 | 0.156447 | 0.008027 | 0.092309 | 0.006708 |
| Cubic splines | 0.61345 | 0.011663 | -0.00243 | 0.012498 | 0.027026 | 0.003117 | 0.039259 | 0.004078 | 0.018557 | 0.003913 | 0.116685 | 0.005087 | 0.156447 | 0.008027 | 0.092309 | 0.006708 |
| Multivariable linear | 0.613148 | 0.012072 | -0.00212 | 0.012623 | 0.027849 | 0.002846 | 0.039259 | 0.004078 | 0.018557 | 0.003913 | 0.121776 | 0.005082 | 0.156447 | 0.008027 | 0.092309 | 0.006708 |
| Two-part | 0.609261 | 0.010897 | 0.001762 | 0.012843 | 0.02935 | 0.002816 | 0.040367 | 0.004112 | 0.018802 | 0.003385 | 0.121792 | 0.005056 | 0.160197 | 0.007961 | 0.085041 | 0.005874 |
| Tobit | 0.626805 | 0.011982 | -0.01578 | 0.013453 | 0.02914 | 0.00314 | 0.038671 | 0.004059 | 0.020012 | 0.004363 | 0.124381 | 0.005341 | 0.154471 | 0.0081 | 0.095579 | 0.007577 |
| ALDVMM | 0.620467 | 0.01333 | -0.00889 | 0.014128 | 0.029695 | 0.003054 | 0.041162 | 0.004858 | 0.01872 | 0.003822 | 0.125099 | 0.005411 | 0.158241 | 0.009545 | 0.093378 | 0.006576 |
| Ordered logistic | 0.676157 | 0.012157 | -0.06458 | 0.010662 | 0.033071 | 0.003695 | 0.045175 | 0.005252 | 0.021477 | 0.003917 | 0.118331 | 0.006497 | 0.150937 | 0.009197 | 0.087113 | 0.007695 |
| SUR | 0.635928 | 0.012344 | -0.02435 | 0.011304 | 0.02885 | 0.002967 | 0.039573 | 0.004248 | 0.018576 | 0.00351 | 0.112525 | 0.005359 | 0.144461 | 0.007991 | 0.081952 | 0.006796 |

## Model residuals

Scatter plots of EQ-5D health index residuals (observed-predicted) over the observed EQ-5D health index for all models.


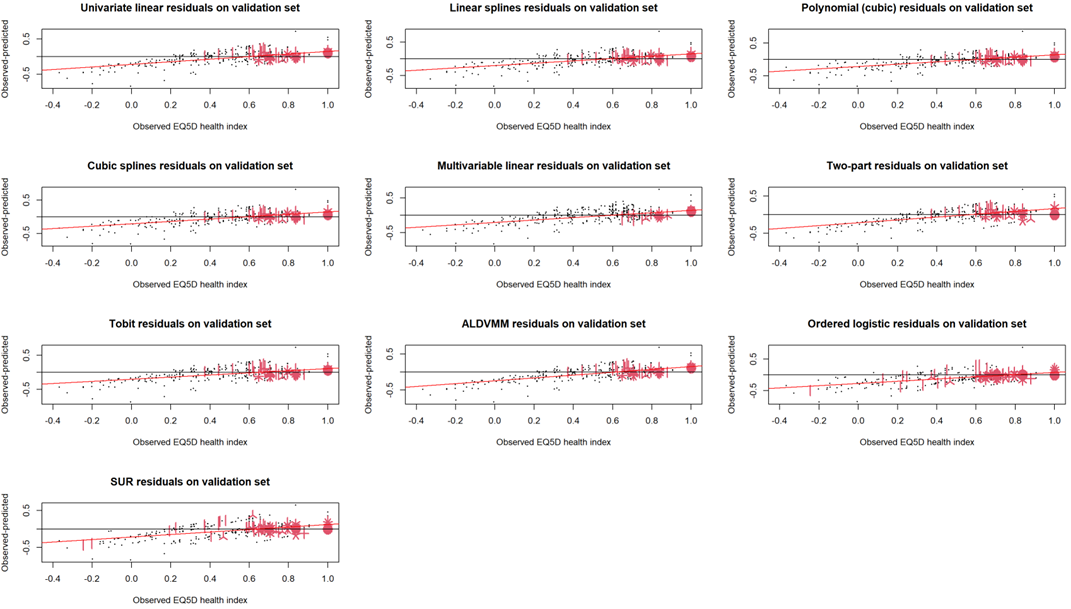

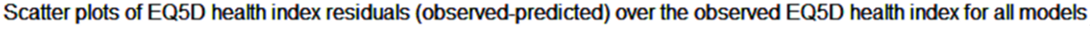


Performance evaluated on the testing sample of the first training-testing (70:30) sample split. The straight red lines demonstrate the correlation between the residuals and the observed EQ-5D health index values. Multiple identical observations depicted by red ‘sunflower’ icons with different numbers of crossing lines. Oxford Shoulder Score (OSS).

## Calibration plots- performance across tenths

Performance metrics are reported for the first training-testing (70:30) sample split.

Training sample- Tenths of predicted EQ-5D index


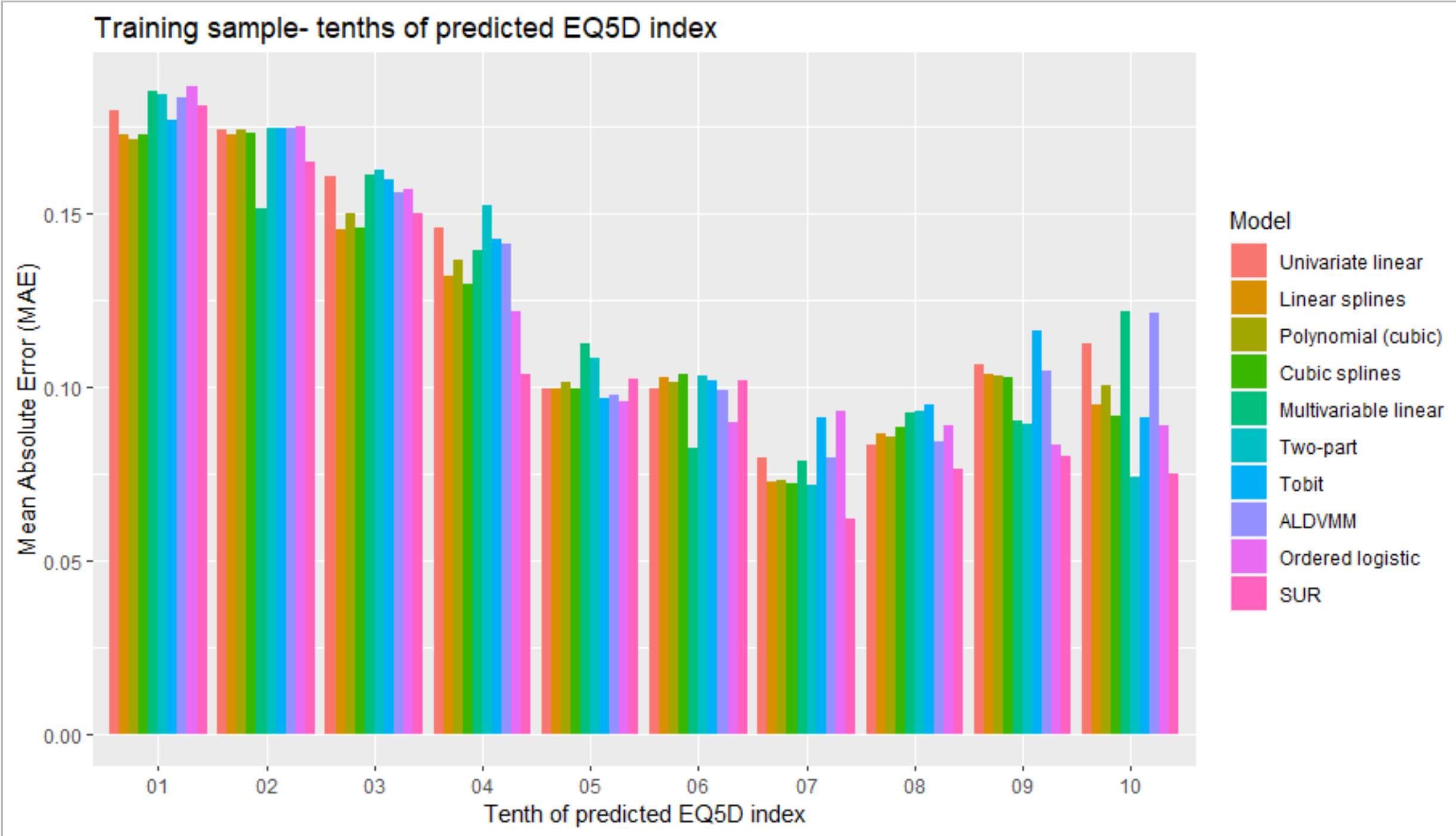


Training sample- Tenths of observed OSS score


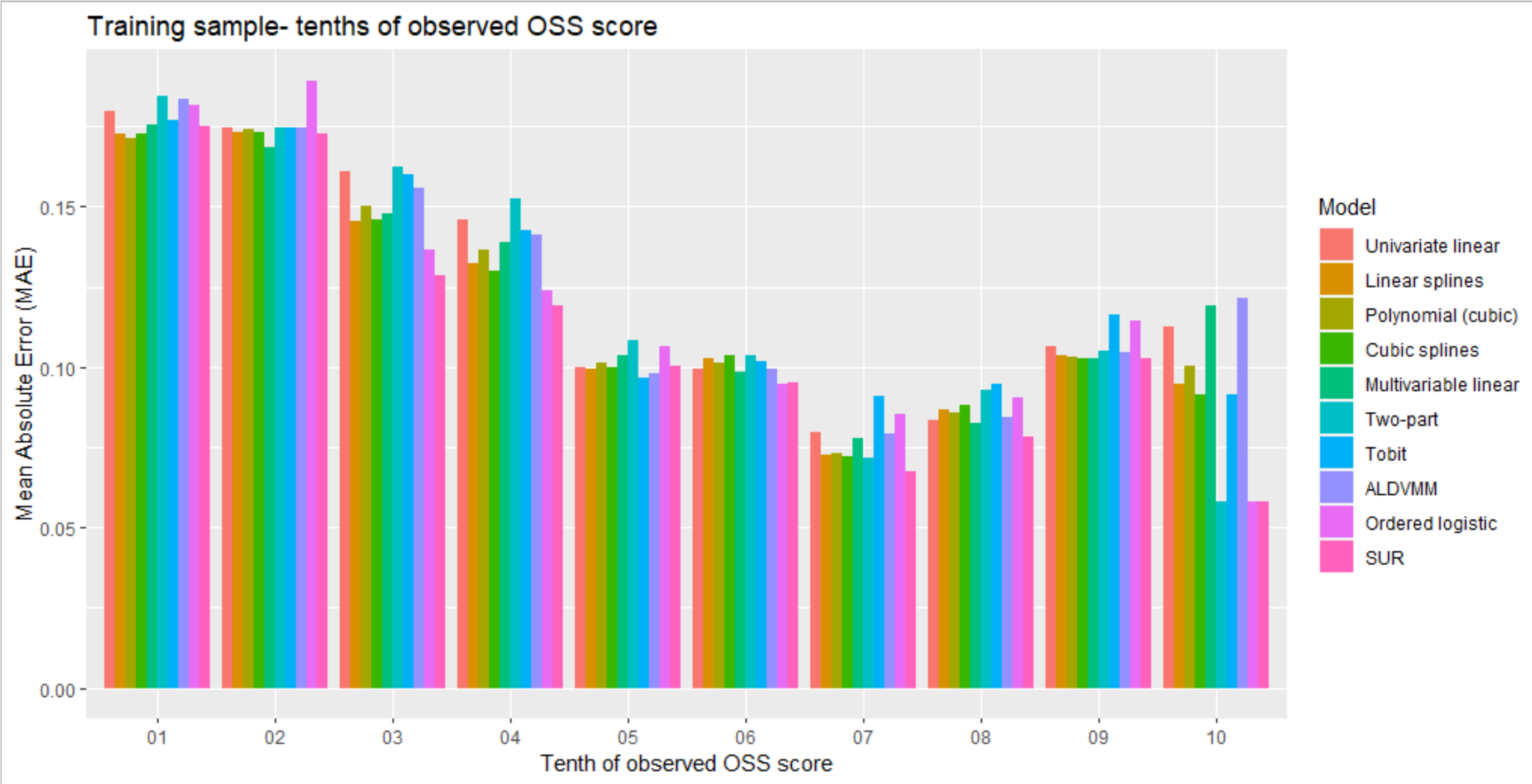


Testing sample- Tenths of predicted EQ-5D index


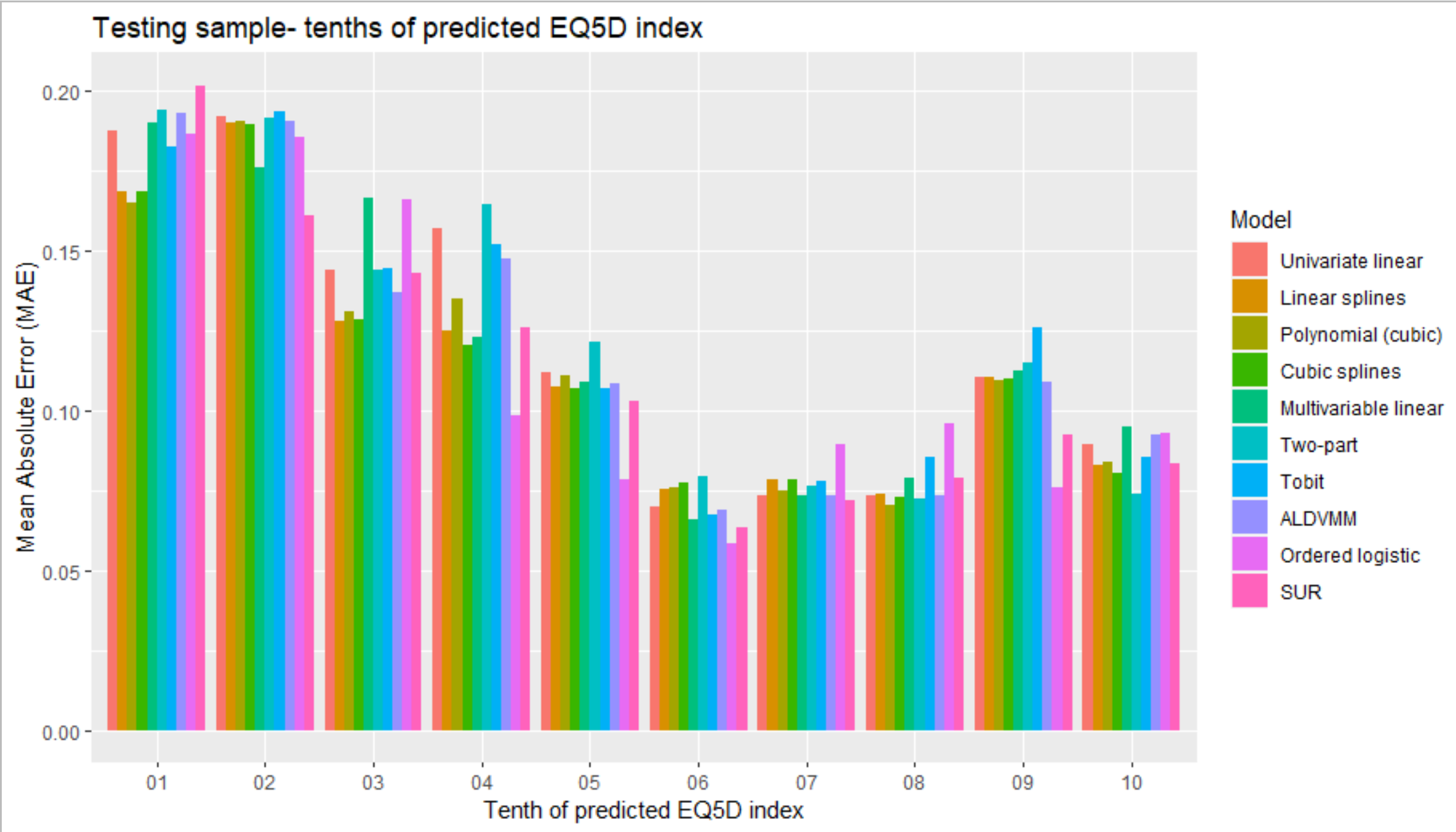


Testing sample- Tenths of observed OSS score


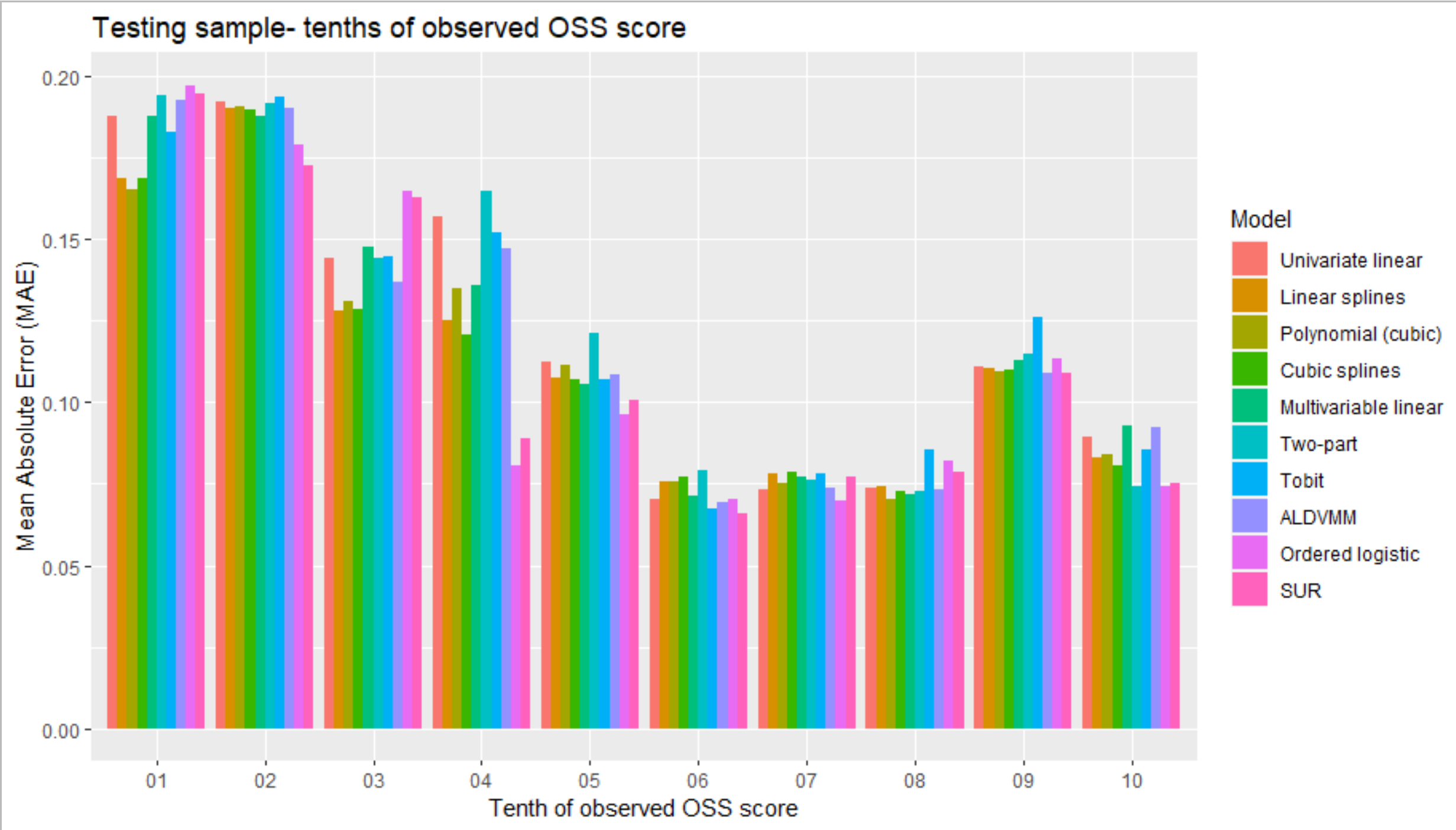


# Section 6: References

1. Petrou S, Rivero-Arias O, Dakin H, et al. The MAPS Reporting Statement for Studies Mapping onto Generic Preference-Based Outcome Measures: Explanation and Elaboration. *PharmacoEconomics*. Published online 2015. doi:10.1007/s40273-015-0312-9

2. Salmerón R, García CB, García J. Variance Inflation Factor and Condition Number in multiple linear regression. *Journal of Statistical Computation and Simulation*. Published online 2018. doi:10.1080/00949655.2018.1463376

3. Austin PC, Escobar M, Kopec JA. The use of the Tobit model for analyzing measures of health status. *Quality of Life Research*. Published online 2000. doi:10.1023/A:1008938326604

4. Sullivan PW. Are utilities bounded at 1.0? Implications for statistical analysis and scale development. *Medical Decision Making*. Published online 2011. doi:10.1177/0272989X11400755

5. McLachlan GJ, Lee SX, Rathnayake SI. Finite mixture models. *Annual Review of Statistics and Its Application*. Published online 2019. doi:10.1146/annurev-statistics-031017-100325

6. Hernández Alava M, Wailoo A. Fitting adjusted limited dependent variable mixture models to EQ-5D. *Stata Journal*. Published online 2015. doi:10.1177/1536867x1501500307

7. Brant R. Assessing Proportionality in the Proportional Odds Model for Ordinal Logistic Regression. *Biometrics*. Published online 1990. doi:10.2307/2532457

8. Gray AM, Rivero-Arias O, Clarke PM. Estimating the association between SF-12 responses and EQ-5D utility values by response mapping. *Medical Decision Making*. Published online 2006. doi:10.1177/0272989X05284108

9. Zellner A. An Efficient Method of Estimating Seemingly Unrelated Regressions and Tests for Aggregation Bias. *Journal of the American Statistical Association*. Published online 1962. doi:10.1080/01621459.1962.10480664

10. Henningsen A, Hamann JD. Systemfit: A package for estimating systems of simultaneous equations in R. *Journal of Statistical Software*. Published online 2007. doi:10.18637/jss.v023.i04
